# Financial toxicity of cancer treatment in India: towards closing the cancer care gap

**DOI:** 10.3389/fpubh.2023.1065737

**Published:** 2023-06-19

**Authors:** Shankar Prinja, Jyoti Dixit, Nidhi Gupta, Anushikha Dhankhar, Amal Chandra Kataki, Partha Sarathi Roy, Nikita Mehra, Lalit Kumar, Ashish Singh, Pankaj Malhotra, Aarti Goyal, Kavitha Rajsekar, Manjunath Nookala Krishnamurthy, Sudeep Gupta

**Affiliations:** ^1^Department of Community Medicine and School of Public Health, Post Graduate Institute of Medical Education and Research (PGIMER), Chandigarh, India; ^2^Department of Radiation Oncology, Government Medical College and Hospital, Chandigarh, India; ^3^Dr. B. Booroah Cancer Institute, Guwahati, India; ^4^Department of Medical Oncology, Adyar Cancer Institute, Chennai, India; ^5^Department of Medical Oncology, All India Institute of Medical Sciences (AIIMS), New Delhi, India; ^6^Department of Medical Oncology, Christian Medical College, Vellore, India; ^7^Department of Clinical Hematology and Medical Oncology, Post Graduate Institute of Medical Education and Research (PGIMER), Chandigarh, India; ^8^Department of Health Research, Ministry of Health and Family Welfare, New Delhi, India; ^9^Department of Medical Oncology, Tata Memorial Centre, Mumbai, India

**Keywords:** financial toxicity, catastrophic health expenditure, impoverishment, direct out of pocket expenditure, indirect cost due to loss of productivity, cancer, outpatient care, hospitalization

## Abstract

**Background:**

The rising economic burden of cancer on patients is an important determinant of access to treatment initiation and adherence in India. Several publicly financed health insurance (PFHI) schemes have been launched in India, with treatment for cancer as an explicit inclusion in the health benefit packages (HBPs). Although, financial toxicity is widely acknowledged to be a potential consequence of costly cancer treatment, little is known about its prevalence and determinants among the Indian population. There is a need to determine the optimal strategy for clinicians and cancer care centers to address the issue of high costs of care in order to minimize the financial toxicity, promote access to high value care and reduce health disparities.

**Methods:**

A total of 12,148 cancer patients were recruited at seven purposively selected cancer centres in India, to assess the out-of-pocket expenditure (OOPE) and financial toxicity among cancer patients. Mean OOPE incurred for outpatient treatment and hospitalization, was estimated by cancer site, stage, type of treatment and socio-demographic characteristics. Economic impact of cancer care on household financial risk protection was assessed using standard indicators of catastrophic health expenditures (CHE) and impoverishment, along with the determinants using logistic regression.

**Results:**

Mean direct OOPE per outpatient consultation and per episode of hospitalization was estimated as ₹8,053 (US$ 101) and ₹39,085 (US$ 492) respectively. Per patient annual direct OOPE incurred on cancer treatment was estimated as ₹331,177 (US$ 4,171). Diagnostics (36.4%) and medicines (45%) are major contributors of OOPE for outpatient treatment and hospitalization, respectively. The overall prevalence of CHE and impoverishment was higher among patients seeking outpatient treatment (80.4% and 67%, respectively) than hospitalization (29.8% and 17.2%, respectively). The odds of incurring CHE was 7.4 times higher among poorer patients [Adjusted Odds Ratio (AOR): 7.414] than richest. Enrolment in PM-JAY (CHE AOR = 0.426, and impoverishment AOR = 0.395) or a state sponsored scheme (CHE AOR = 0.304 and impoverishment AOR = 0.371) resulted in a significant reduction in CHE and impoverishment for an episode of hospitalization. The prevalence of CHE and impoverishment was significantly higher with hospitalization in private hospitals and longer duration of hospital stay (*p* < 0.001). The extent of CHE and impoverishment due to direct costs incurred on outpatient treatment increased from 83% to 99.7% and, 63.9% to 97.1% after considering both direct and indirect costs borne by the patient and caregivers, respectively. In case of hospitalization, the extent of CHE increased from 23.6% (direct cost) to 59.4% (direct+ indirect costs) and impoverishment increased from 14.1% (direct cost) to 27% due to both direct and indirect cost of cancer treatment.

**Conclusion:**

There is high economic burden on patients and their families due to cancer treatment. The increase in population and cancer services coverage of PFHI schemes, creating prepayment mechanisms like E-RUPI for outpatient diagnostic and staging services, and strengthening public hospitals can potentially reduce the financial burden among cancer patients in India. The disaggregated OOPE estimates could be useful input for future health technology analyses to determine cost-effective treatment strategies.

## Introduction

Cancer accounted for nearly 10 million deaths in the year 2020, or nearly one in six deaths (1, 2). Globally, there were 23.6 million new cases of cancer as per World Health Organization (WHO) report (1, 2). High-income countries recorded the highest incidence rates, however, deaths due to cancer were majorly reported from low- and middle-income countries owing to poor accessibility to quality and timely treatment (3). The GLOBACON 2018 report estimated 1 million new cancer cases and 0.7 million deaths in India annually, which is estimated to increase to 2 million cases and 1 million deaths by 2040 (4).

The rising prevalence of cancer further increases the stress on already burdened healthcare system, and also imposes physical, psychosocial, and financial strain on the patients and their families (5–7). The costly and intensive diagnostic and treatment modalities used for cancer are often financially taxing for payers—be it the government or households. Low health insurance coverage, and high reliance on out-of-pocket payments further increase the financial toxicity associated with cancer treatment (5–7). High out-of-pocket expenditure (OOPE) leads to financial catastrophe, deeper debts, and impoverishment of the households with patients undergoing cancer treatment (5–7). The odds of impoverishment due to high OOPE are six times higher for cancer treatment than that due to infectious diseases in India (8).

In view of this, it is important to reduce socioeconomic inequalities in access to cancer care by increasing the provision of quality, affordable, and accessible healthcare services (9). In 2018, the government of India launched its publically funded health insurance program, “Ayushman Bharat Pradhan Mantri Jan Arogya Yojana” (AB PM-JAY), which provides access to cashless surgical, medical and radiation therapy for cancers through its vast network of more than 28,000 empanelled hospitals (10).

Several studies have attempted to estimate the economic burden of cancer in the past (11–19). However, there were certain methodological limitations in published studies. Most of the existing studies are single-centric and have small sample sizes (13, 19–21). Most of the studies have used total household consumption expenditure (22, 23) as a measure to compute catastrophic health expenditure (CHE). However, when total expenditure of the household is regarded as the denominator, CHE is defined relative to the health payments budget. The potential issue is that poor in low income countries may have low budget share and most of the resources are spent on meeting basic household needs and small share is spared for spending on health. Therefore, the households who cannot pay for health services are not taken into account in this definition. Few studies were also found to use household income as a measure for assessment of CHE (24, 25). However, given the kind of economy that prevails in India, there is lot of under-reporting of income and true economic impact can be assessed without reliable estimates on household’s capacity to pay. In view of this, the present study has used the standardised approach for eliciting financial toxicity and used household’ capacity to pay as an indicator to measure CHE. The household’s capacity to pay was estimated by subtracting subsistence expenditure from total household consumption expenditure.

Certain inconsistencies were also observed in the range of medical services for which OOPE was recorded (e.g., cost of surgery or medicines), type of cost included (direct medical/non-medical/both), cancer categories (most studies focussed on single cancer site, i.e., breast, cervical or prostate cancer etc.), and type of healthcare setting (e.g., single-centre studies from a tertiary care hospital/private healthcare facility etc.). Although the 75th round of National Sample Survey on Social Consumption on Health in India reports the economic burden of different ailment categories including cancer, the sample barely includes 1,751 cancer patients (26). This sample is insufficient to undertake stratified analyses by type of cancer, treatment or disease severity. Similarly, it is not powered enough to elicit the determinants of financial hardship. Moreover, it is cross-sectional data collection which does not provide a comprehensive assessment of OOPE annually for cancer treatment of both outpatient and inpatient care per patient.

We undertook the present study to provide comprehensive evidence on the economic burden of cancer by collecting primary data on direct out-of-pocket expenditure (OOPE) from a large representative sample of cancer patients (*N* = 12,148) drawn from different regions of India. We report estimates on direct out-of-pocket expenditure incurred on outpatient and hospitalized treatment at overall level, and by site, stage, response, and type of treatment. In a subsample of 3,251 patients, we have also assessed indirect costs borne by the patient due to outpatient treatment and hospitalization. We also measured financial toxicity in terms of extent of CHE and impoverishment among patients seeking cancer care in India using the standardised methodology, as well as assessed the determinants of financial toxicity due to only direct costs and total societal costs (direct plus indirect costs).

## Methodology

A cross-sectional study was conducted at selected seven health care facilities providing cancer care across six states in India. The detailed methodology of the study is published in the protocol paper (27).

### Selection of healthcare facilities

**Figure 1:**
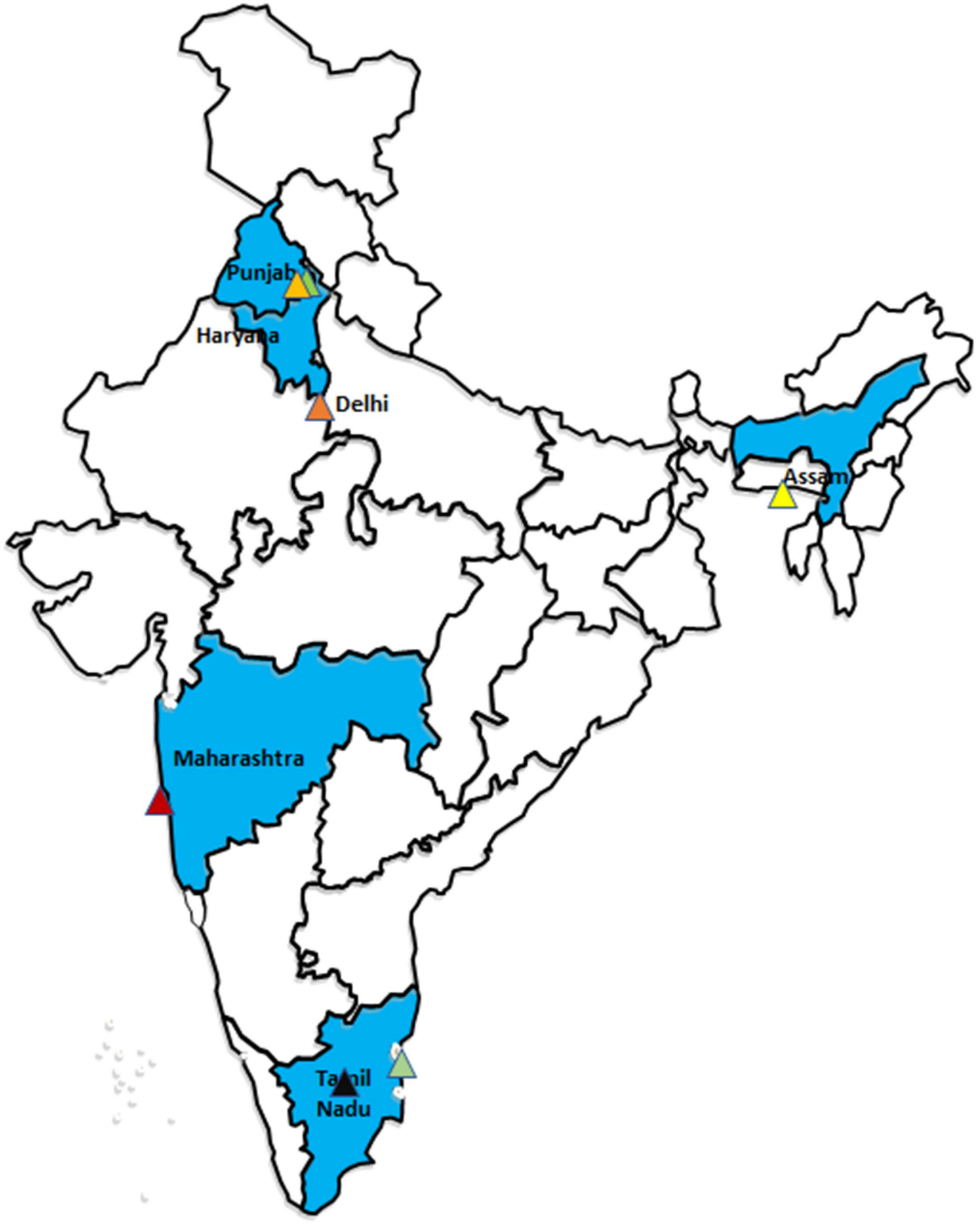
Selected study regions.

A multi-stage stratified sampling technique was followed for recruiting cancer patients. In the first stage, the states were stratified into three categories based on the epidemiological transition level (ETL) using the ratio of disability adjusted life years (DALYs) lost as a result of communicable, maternal, neonatal and nutritional diseases, to the DALYs lost due to non-communicable diseases (including cancer and injuries) (28). Among high ETL states (ratio: less than 0·31), Chandigarh (Punjab) and Tamil Nadu were randomly selected. Similarly, among middle (ratio: 0·31–0·55) and low (ratio: 0·56–0·75) ETL states, Delhi & Maharashtra, and Assam were selected, respectively (Figure 1).

At the second stage, seven health-care facilities that catered to largest volume of oncology patients in these sites were purposively selected. Two of these cancer centres contribute to the highest volume of patients treated under India’s largest national insurance programme, AB PM-JAY. At the third stage, the total desired sample of patients to be recruited at each facility was achieved using systematic random sampling technique (Supplementary Table S1, Supplementary Appendix S1).

### Patient recruitment

#### Sampling technique

The patients were recruited prospectively between October 2020 to March 2022 at outpatient and inpatient departments of the selected health care facilities. For facilities with common clinics for all types of cancer, systematic random sampling was used to recruit patients with a sampling interval based on mean daily number of patients. For centres with cancer clinics representing different disease management groups (DMGs), PPS method was used to determine the sample size of patients to be recruited at each DMG.

#### Sample size

Considering the mean OOPE of ₹ 57,232 with standard deviation of ₹ 86,871 at 95% CI and 5% margin of error, a sample size of 1,536 was estimated for each of the seven healthcare facilities (29). Taking non-response rate of 10%, the minimum sample size at each centre was estimated as 1,690. We also estimated sample size requirement for other two primary endpoints of the study-CHE and impoverishment. As the OOPE estimation yielded the highest sample size, the minimum of 1,690 patients were interviewed for assessment of OOPE and financial toxicity at each participating centre. Overall, we recruited 12,148 cancer patients (9,787 outpatients and 5,095 inpatients) (27).

#### Inclusion criteria

Patients of all age groups and gender with an established cancer diagnosis who sought outpatient and hospitalized treatment at selected health care facilities for any cancer type or stage were eligible to be included in the study. Case definitions used for recruiting patients are described in Supplementary Appendix S1.

#### Data collection

A written informed consent was obtained and data on OOPE were collected from all study participants above 18 years of age and from parents/guardians/proxy respondents for minors. A pretested structured interview schedule was used to collect information on socio-demographic characteristics, household consumption expenditure, clinical data, and OOPE (Supplementary Appendices S2, S3).

Newly diagnosed and on-treatment patients who sought outpatient care within last 30 days were recruited and interviewed on the same day for recording direct medical (consultation fee, diagnostic test charges, medicine charges, user fee, etc.) and non-medical (travelling cost, boarding/lodging cost, cost of food, etc.) OOPE incurred since last visit. However, for cases who sought care more than 30 days ago, telephonic interviews were conducted on the 15th day following recruitment to elicit the data. For ensuring high response rate on telephonic interviews, a minimum of 2–3 contact numbers were recorded (30, 31). Details of any episodes of hospitalization during last 1 year among patients who were recruited in outpatient setting were also elicited. We also recruited patients who were admitted due to cancer and data on expenditures incurred on each day of hospitalization till discharge was collected. The invoices of expenditure incurred were obtained to ensure accuracy of data. The average number of hospitalizations (1.7) observed in patients recruited at outpatient settings was used to compute annual OOPE on hospitalization among patients recruited in inpatient setting as OOPE elicited using patient interviews was only for single episode of hospitalization.

In a subsample of 3,251 patients (2,577 outpatients and 674 hospitalized cases) drawn from five states data of India using systematic random sampling technique, we have also assessed the indirect costs due to loss of productivity in addition to direct costs incurred on cancer treatment. We have used the human capital approach for estimation of indirect costs (32). The morbidity element of the indirect costs was captured by enquiring patient and their caregivers about the lost hours of their productive time due to cancer treatment for 10 activities—household work, childcare activities, professional work, voluntary work, social work, seeking work, attending school, physical workout, leisure activities and others (Supplementary Appendix S4). These hours reflect the time that could have been spent by patients or caregivers on various activities mentioned above. Further, the number of hours of paid activities delegated to another individual by the patient or caregiver was also recorded. Indirect cost for a patient was then calculated as:


Indirect cost=Total number of work days×daily wage rates

We have considered average time duration of work as 8 h based on International Labor Organization 2017 report (33). The total number of work days foregone by the patient was calculated by dividing total hours forgone in a month with ideal work hours per day (8 h). Further, daily wage rates were computed using *per capita* consumption expenditure. Total household consumption expenditure was divided by equivalent household size to compute *per capita* consumption expenditure.


$$eqsize_{h}=hhsize_{h}^{\beta }$$

where hhsize is the household size, and the value of parameter *β* has been estimated from previous studies based on 59 countries’ household survey data which is equal to 0.56 (34). For caregivers, daily wage rates were computed using self-reported monthly income and, the product of hours forgone and daily wage rates represented the indirect cost. Mean and standard deviation (SD) was computed for indirect cost estimation.

#### Data analysis

The mean OOPE and standard error (SE) were computed for both per episode of hospitalization and outpatient treatment. In addition, total annual OOPE per patient was also estimated by using the following equation:


$$\begin{array}{c} \mathrm{Annual OOPE}=\left[\mathrm{OOPE}\;\mathrm{per}\;\mathrm{OPD}\;\mathrm{episode}\times n_{\mathrm{OPD}}\times 12\right]\\ +\sum_{n=1}^{n}{\mathrm{OOPE}}_{\mathrm{hospitalization}} \end{array}$$

where $n_{\mathrm{OPD}}\;$ = Mean monthly outpatient visits (derived using primary data collected from 1,279 cancer patients).

Proportion of households with OOPE equal to or exceeding 40% of household’s capacity to pay or non-subsistence spending were considered to have experienced CHE (35, 36). Household’s capacity to pay is calculated through subtracting expenditures on basic needs from the total household consumption expenditure (34). The subsistence expenditure (SE_h_) is the minimum requirement to maintain basic life in a society. We have used a food share based poverty line for estimating household subsistence. This poverty line is defined as the mean food expenditure of the household whose food expenditure share of total household expenditure (Exp_h_) is within 45th to 55th percentile of the total sample (35).

Impoverishment was considered when household expenditure was equal to or higher than subsistence spending (Exp_h_ ≥ SE_h_) but lower than subsistence spending net of out of pocket health payments (SE_h_ > Exp_h_-OOPE_h_) (35). Details on components of subsistence expenditure and recall period can be found in the Supplementary Appendix S2 of the manuscript.

This was followed by logistic regression model for estimation of parameters. The logistic regression is used to analyse the association between the catastrophic and impoverishment while controlling for potential confounders. Logistic regression is actually an extension of linear regression rather than modelling a linear relationship between the independent variables (Xi) and the probability of the outcome. The logistic regression equation is assumed to be


(1) $$t=\mathrm{Ln}\left(\frac{p}{1-p}\right)=\beta_{0}+\beta_{1}X_{1}+\beta_{2}X_{2}+\cdots \beta_{k}X_{k}$$

where, t is the log-odds, X_i is the value of the ith predictor, β_i represent parameters of model, i = 1, 2, …k.

Independent variables comprised of socio-demographic variables including age, gender, area of residence, level of education, income status, type of financial benefit scheme and, clinical characteristics namely type of cancer, type of treatment, cancer stage, type of response, line of treatment and adverse effects.


$$\mathrm{Catastrophic health expenditure}=\{\begin{array}{c} 1\;\mathrm{if}\;\frac{\mathrm{OOP}}{\mathrm{CTP}}\geq 0.4\\ 0\;\mathrm{if}\;\frac{\mathrm{OOP}}{\mathrm{CTP}}<0.4 \end{array}$$

$$\mathrm{Impoverishment}=\{\begin{array}{c} 1\;\mathrm{if}\left(\;\exp\geq \mathrm{se}\right)\mathrm{and}\;\left(\exp-\mathrm{oop}<\mathrm{se}\right)\;\\ 0\;\mathrm{if}\;\left(\;\exp\geq \mathrm{se}\right)\mathrm{and}\;\left(\exp-\mathrm{oop}\geq \mathrm{se}\right) \end{array}$$

where exp. represents consumption expenditure, SE is subsistence expenditure, OOP is out of pocket expenditure and CTP represents household capacity to pay. In addition, bivariate analysis was done to assess the associations between OOPE and socio-demographic as well as clinical characteristics (Supplementary Tables S1–S3, Supplementary Appendix S5). The factors influencing the OOPE were also assessed using regression analyses (Supplementary Tables S4, S5, Supplementary Appendix S5).

## Ethical considerations

The study complies with the Declaration of Helsinki and an ethical approval to undertake the study was obtained from Institute Ethics Committee of the Postgraduate Institute of Medical Education and Research (PGIMER), Chandigarh with reference number IEC-03/20202-1565. Informed written consent was obtained from all the study participants.

## Results

A total of 9,897 patients were recruited in the outpatient setting. Out of these, 2,736 patients reported at least one episode of hospitalization during last 1 year. In addition, a total of 2,361 patients were recruited while being hospitalized in study centres. The expenditures incurred on all episodes of hospitalization during 1 year time period was used to compute indicators of financial risk protection.

### Socio-demographic profile of cancer patients

Majority of the patients seeking cancer treatment, were in the age group of 45–60 years (40.5% outpatients and 41.5% hospitalized patients), belonged to rural area (65% outpatient cases and 62.7% hospitalized cases) and were females (58.8% hospitalized and 58.3% outpatient patients). Approximately 60% of the patients seeking outpatient treatment and 62.8% hospitalised patients were found to be covered under some health insurance schemes. Nearly, 10.3% outpatient cases and 13.3% hospitalized cases were enrolled under AB PM-JAY; 33% were covered under state-sponsored health insurance schemes (including both AB PM-JAY and other state health insurance schemes). The sociodemographic profile of cancer patients is summarised in Table 1.

**Table 1:** Sociodemographic profile of cancer patients.

Sociodemographic characteristics	Outpatient cases		Hospitalized cases	
	*N*	%	*N*	%
*Age groups (in years)*				
0–15	311	3.2	74	2.7
16–30	778	7.9	229	8.4
31–45	2,559	26.1	747	27.3
45–60	3,965	40.5	1,135	41.5
Above 60	2,174	22.2	551	20.1
*Gender*				
Male	4,078	41.7	1,127	41.2
Female	5,709	58.3	1,609	58.8
*Area of residence*				
Urban	3,381	34.5	972	35.5
Rural	6,269	64.1	1,715	62.7
Slum	137	1.4	49	1.8
*Education*				
No education	2,124	21.7	594	21.7
Primary and middle	3,435	35.1	949	34.7
Up-to senior secondary	2,942	30.1	804	29.4
Graduation and above	1,286	13.1	389	14.2
*Wealth quintile*				
Poorest	1958	20	499	18.2
Poor	1960	20	492	18.0
Middle	1956	20	550	20.1
Rich	1956	20	584	21.3
Richest	1957	20	611	22.3
*Marital status*				
Unmarried	895	9.1	233	8.5
Married	7,823	79.9	2,173	79.4
Separated/divorced	66	0.7	22	0.8
Widow/widower	1,003	10.2	308	11.3
*Health insurance*				
AB-PMJAY	1,009	10.3	365	13.3
State government sponsored^a^	3,230	33	905	33.1
Patient support groups (Philanthropist/NGO)	618	6.3	167	6.1
Social insurance scheme	568	5.8	191	7.0
Private health insurance	369	3.8	90	3.3
Not covered	3,993	40.8	1,018	37.2
Total	9,787		2,736	

a State government sponsored category includes patients enrolled in AB-PMJAY and other state health insurance schemes.

### Direct OOPE on cancer treatment

**Figure 2:**
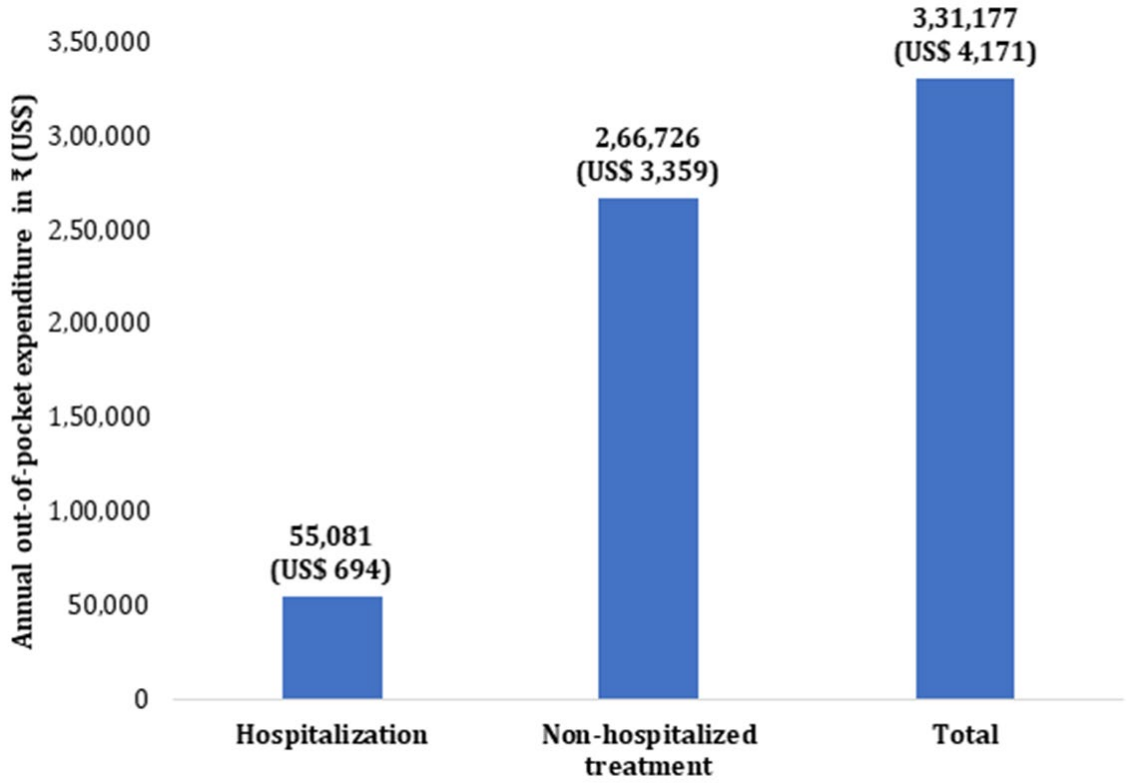
Annual out-of-pocket expenditure on cancer treatment.

**Figure 3:**
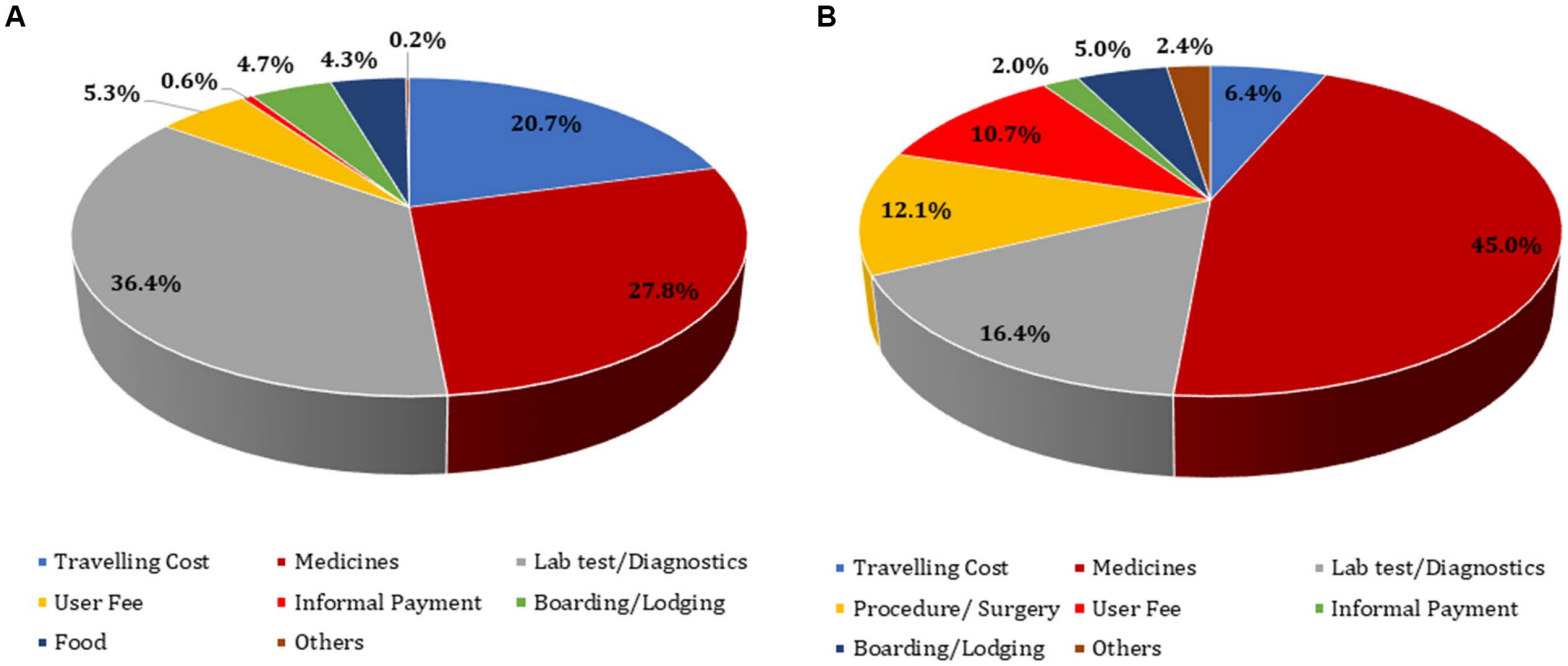
Components of out-of-pocket expenditure on cancer treatment.

Per visit mean OOPE on outpatient cancer treatment was computed as ₹ 8,053 [95% CI: ₹ 7,772–8,335]. Taking into account the average number of visits per month for outpatient treatment as 2.76 (based on actual utilization pattern), mean monthly direct OOPE incurred on outpatient treatment was estimated as ₹ 22,227 (95% CI: ₹ 21,450–23,004). The estimated OOPE per episode of hospitalization was ₹ 39,085 (95% CI: ₹ 36,431–41,738). The total annual direct OOPE on cancer treatment was estimated as ₹ 331,177 (95% CI: ₹ 320,142–342,212) (Figure 2). The diagnostics (36.4%) and medicines (27.8%) were major contributors of OOPE for outpatient treatment. For hospitalized treatment, medicines (45%), diagnostics (16.4%), and procedure/surgery (12.1%) were major contributors of OOPE (Figure 3). Various sources of financing OOPE are detailed in Appendix S5 (Supplementary Figure S1).

### Financial toxicity due to cancer treatment

The overall prevalence of CHE was found to be 80.4% due to outpatient treatment and 29.8% as a result of cancer-related hospitalization. The overall prevalence of impoverishment was 67% due to outpatient cancer treatment and 17.2% due to hospitalization.

### Catastrophic health expenditure

**Figure 4:**
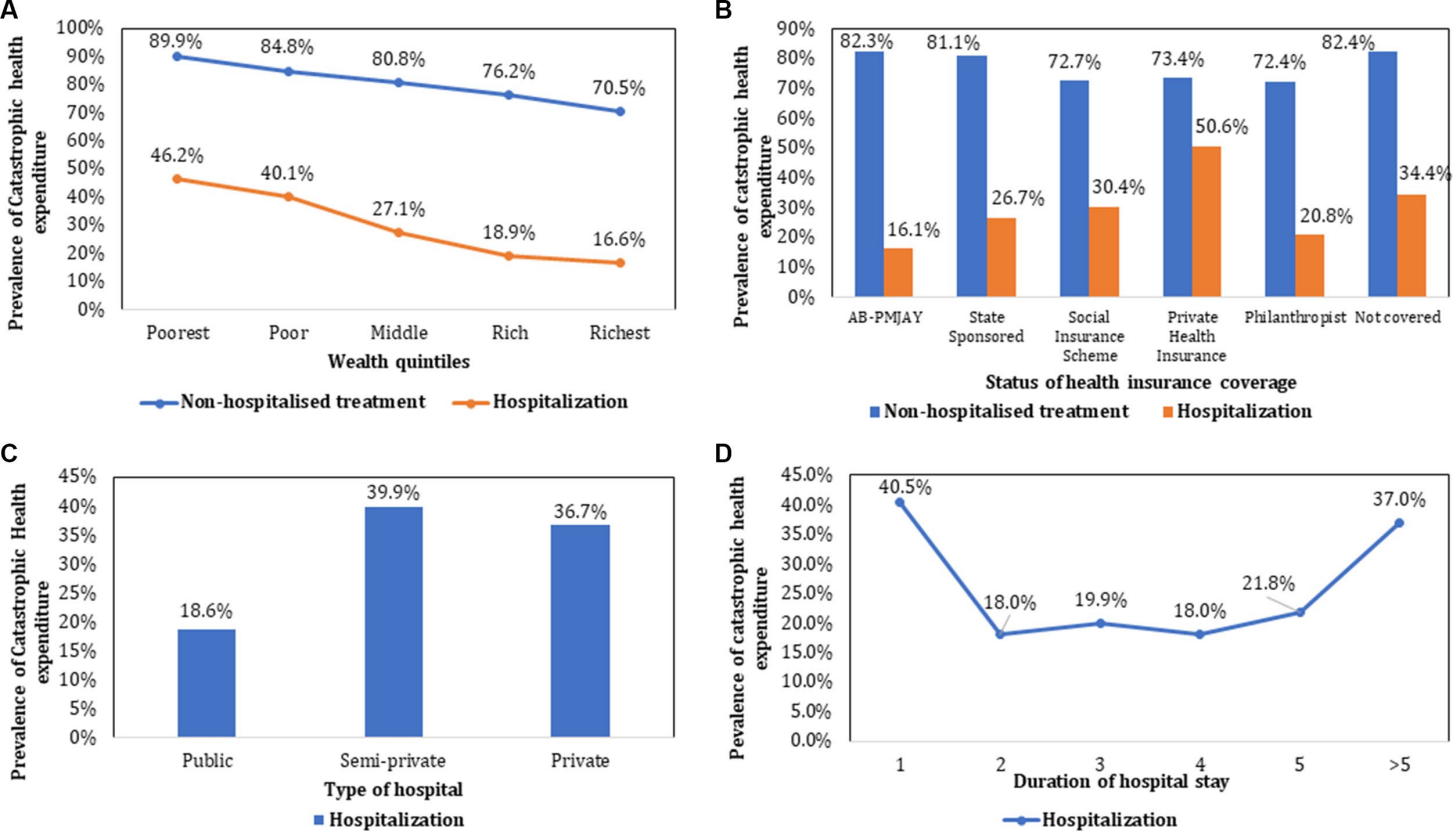
Prevalence of catastrophic health expenditure stratified by socio-demographic and clinical characteristics.

For both outpatient treatment and hospitalisation, the prevalence of CHE showed a declining trend from poorest to the richest income quintiles as shown in Figure 4. As compared to CHE for hospitalization among those who were not covered by any health insurance (34.4%), the prevalence was lower among those covered under AB PM-JAY (16.1%) which was statistically significant (*p* < 0.001). Similarly, CHE was significantly higher for those admitted in private hospitals (36.7%), compared to public hospitals (18.6%). The prevalence of CHE among different subgroups (stratified by socio-demographic and clinical characteristics) of cancer patients seeking outpatient and hospitalized treatment has been summarized in Tables 2, 3.

**Table 2:** Prevalence and determinants of catastrophic health expenditure and impoverishment due to cancer-related outpatient treatment.

Patient characteristics	No. of patients *N* (%)	Prevalence of CHE (%)	CHE	*p*-value	Prevalence of impoverishment (%)	Impoverishment	*p*-value
			Adjusted odds ratio (95% CI)			Adjusted odds ratio (95% CI)	
*Age groups (in years)*							
0–15	311 (3.2%)	85.2	1.007 (0.999, 1.015)	0.102	66.8	1.007 (1, 1.015)	0.059
16–30	778 (7.9%)	84.7			65.1		
31–45	2,559 (26.1%)	79.3			60.2		
45–60	3,965 (40.5%)	79.1			58.8		
Above 60	2,174 (22.2%)	81.9			64.2		
*Gender*							
Male	4,078 (41.7%)	83.3	Reference		65.0	Reference	
Female	5,709 (58.3%)	78.4	0.948 (0.767, 1.173)	0.625	58.3	0.881 (0.727, 1.069)	0.200
*Area of residence*							
Urban	3,381 (34.5%)	70.4	Reference		52.6	Reference	
Rural	6,269 (64.1%)	86.0	3.665 (2.987, 4.498)	<0.001	66.3	2.451 (1.996, 3.01)	<0.001
Slum	137 (1.4%)	72.3	1.146 (0.58, 2.264)	0.694	50.0	1.125 (0.554, 2.284)	0.745
L*evel of education*							
No education	2,124 (21.7%)	83.1	Reference		61.8	Reference	
Primary and middle	3,435 (35.1%)	81.1	0.911 (0.703, 1.18)	0.480	62.9	1.185 (0.936, 1.502)	0.159
Up-to senior secondary	2,942 (30.1%)	79.7	0.943 (0.714, 1.245)	0.678	61.6	1.326 (1.031, 1.706)	0.028
Graduation and above	1,286 (13.1%)	75.5	1.334 (0.929, 1.915)	0.119	55.4	1.381 (0.998, 1.911)	0.051
*Wealth quintile*							
Poorest	1958 (20%)	89.9	10.959 (7.636, 15.728)	0.000	86.9	46.78 (21.101, 103.71)	0.000
Poor	1960 (20%)	84.8	4.136 (3.068, 5.576)	0.000	80.7	12.014 (8.908, 16.203)	0.000
Middle	1956 (20%)	80.8	2.201 (1.657, 2.924)	0.000	65.5	3.709 (2.902, 4.742)	0.000
Rich	1956 (20%)	76.2	1.243 (0.955, 1.616)	0.105	54.2	1.562 (1.241, 1.966)	0.000
Richest	1957 (20%)	70.5	Reference		44.6	Reference	
*Type of financial benefit scheme*							
Not covered	3,993 (40.8%)	82.4	Reference		64.9	Reference	
AB-PMJAY	1,009 (10.3%)	82.3	0.631 (0.429, 0.93)	0.020	61.1	0.597 (0.433, 0.825)	0.002
State sponsored^a^	3,230 (33%)	81.1	0.5 (0.393, 0.635)	<0.001	60.6	0.503 (0.407, 0.621)	<0.001
Social insurance scheme	568 (5.8%)	72.7	0.646 (0.402, 1.038)	0.071	51.0	0.4 (0.261, 0.615)	<0.001
Private health insurance	369 (3.8%)	73.4	0.829 (0.468, 1.469)	0.521	52.1	0.552 (0.322, 0.947)	0.031
Philanthropist	618 (6.3%)	72.4	0.258 (0.17, 0.391)	<0.001	50.2	0.434 (0.261, 0.72)	0.001
*Type of cancer*							
Solid	7,618 (78%)	79.3	Reference		59.4	Reference	
Hematological	2,101 (21.5%)	84.3	2.413 (0.506, 11.502)	0.269	67.1	1.573 (0.467, 5.294)	0.465
CUPS^b^	42 (0.4%)	90.2	0.757 (0.087, 6.606)	0.801	74.1	0.87 (0.136, 5.581)	0.884
*Type of treatment*							
Chemotherapy	4,304 (50.6%)	83.4	Reference		63.0	Reference	
Radiotherapy	347 (4.1%)	83.2	0.878 (0.578, 1.333)	0.542	59.9	0.941 (0.644, 1.376)	0.755
Palliative care	236 (2.8%)	81.8	0.913 (0.484, 1.723)	0.779	63.6	1.347 (0.754, 2.405)	0.314
Surgery	519 (6.1%)	78.6	0.823 (0.579, 1.17)	0.278	60.7	1.204 (0.856, 1.693)	0.287
Combination therapy	913 (10.7%)	76.3	0.887 (0.672, 1.169)	0.394	52.7	0.951 (0.745, 1.214)	0.688
Maintenance therapy	179 (2.1%)	88.8	0.628 (0.084, 4.694)	0.650	70.9	0.822 (0.087, 7.805)	0.865
Diagnostic	97 (1.1%)	90.4	1.249 (0.119, 13.117)	0.853	78.9	3.854 (0.316, 46.978)	0.290
Hormone therapy	238 (2.8%)	62.9	0.326 (0.203, 0.523)	<0.001	43.6	0.412 (0.239, 0.709)	0.001
Others	1,666 (19.6%)	74.2	0.478 (0.306, 0.747)	0.001	53.9	0.556 (0.328, 0.944)	0.030
*Cancer stage*							
Stage I	413 (4.2%)	73.0	Reference		52.3	Reference	
Stage II	1,181 (12.1%)	78.4	1.198 (0.824, 1.741)	0.345	54.9	1.098 (0.751, 1.606)	0.629
Stage III	2,165 (22.1%)	78.1	1.22 (0.868, 1.715)	0.252	58.7	1.298 (0.91, 1.853)	0.150
Stage IV	1,564 (16%)	85.5	1.806 (1.235, 2.639)	0.002	65.4	1.587 (1.09, 2.311)	0.016
*Type of response*							
Progression free survival	2,402 (24.5%)	70.6	Reference		49.5	Reference	
Progressive disease	450 (4.6%)	80.6	1.361 (0.826, 2.244)	0.227	59.9	1.085 (0.681, 1.729)	0.731
Ongoing	5,394 (55.1%)	83.1	1.462 (1.112, 1.922)	0.007	62.4	1.65 (1.247, 2.182)	<0.001
*Line of treatment*							
First line	6,817 (69.7%)	79.2	Reference		58.4	Reference	
Second line	1,146 (11.7%)	79.9	0.874 (0.627, 1.219)	0.428	59.6	1.016 (0.733, 1.408)	0.924
Third line	163 (1.7%)	83.4	1.276 (0.512, 3.178)	0.601	66.2	1.471 (0.642, 3.373)	0.362
Fourth line	20 (0.2%)	75.0	0.271 (0.049, 1.486)	0.133	55.0	0.916 (0.168, 5)	0.919
*Adverse effect*							
Without adverse effect	564 (5.8%)	69.3	Reference		50.1	Reference	
With adverse effect	5,145 (52.6%)	82.9	1.204 (0.815, 1.778)	0.352	62.3	1.333 (0.84, 2.114)	0.222
Total	9,787	80.4			61.1		

a State government sponsored category includes patients enrolled in AB-PMJAY and other state health insurance schemes.

b CUPS-Cancer of Unknown Primary Site.

**Table 3:** Prevalence and determinants of catastrophic health expenditure and impoverishment due to cancer-related hospitalization.

Patient characteristics	Number of patients, *N* (%)	Prevalence of CHE (%)	CHE	*p*-value	Prevalence of impoverishment (%)	Impoverishment	*p*- value
			Adjusted odds ratio (95% CI)			Adjusted odds ratio (95% CI)	
*Age groups (in years)*							
0–15	232 (4.6%)	27.2	1.003 (0.998, 1.009)	0.230	14.8	1.006 (0.998, 1.013)	0.168
16–30	500 (9.8%)	31.2			19.1		
31–45	1,296 (25.4%)	26.9			15.1		
45–60	2020 (39.6%)	30.9			17.8		
Above 60	1,047 (20.5%)	31.0			18.5		
*Gender*							
Male	2,324 (45.6%)	29.1	Reference		17.7	Reference	
Female	2,771 (54.4%)	30.3	1.092 (0.949, 1.258)	0.218	16.8	0.915 (0.755, 1.108)	0.363
*Area of residence*							
Urban	1972 (38.7%)	34.4	Reference		21.5	Reference	
Rural	3,046 (59.8%)	27.0	1.021 (0.875, 1.191)	0.791	14.6	0.971 (0.786, 1.199)	0.783
Slum	77 (1.5%)	23.4	1.038 (0.579, 1.861)	0.900	7.5	0.66 (0.25, 1.741)	0.401
*Level of education*							
No education	972 (19.1%)	22.5	Reference		10.7	Reference	
Primary and middle	1,685 (33.1%)	27.2	1.114 (0.908, 1.367)	0.302	16.5	1.308 (0.965, 1.773)	0.083
Up to senior secondary	1,562 (30.7%)	32.8	1.533 (1.241, 1.894)	0.000	17.5	1.461 (1.07, 1.993)	0.017
Graduation and above	876 (17.2%)	37.4	1.926 (1.506, 2.464)	0.000	24.6	2.483 (1.763, 3.496)	0.000
*Wealth quintile*							
Poorest	1,019 (20%)	46.2	7.414 (5.826, 9.435)	0.000	51.0	28.432 (18.641, 43.367)	0.000
Poor	1,019 (20%)	40.1	5.305 (4.213, 6.681)	0.000	34.2	13.766 (9.94, 19.065)	0.000
Middle	1,019 (20%)	27.1	2.952 (2.335, 3.731)	0.000	15.4	4.866 (3.484, 6.797)	0.000
Rich	1,020 (20%)	18.9	1.576 (1.235, 2.011)	0.000	8.6	2.086 (1.457, 2.987)	0.000
Richest	1,018 (20%)	16.6	Reference		6.0	Reference	
*Type of financial benefit scheme*							
Not covered	1931 (37.9%)	34.4	Reference		19.1	Reference	
AB-PMJAY	634 (12.4%)	16.1	0.426 (0.33, 0.548)	0.000	8.1	0.395 (0.277, 0.565)	0.000
State sponsored^a^	1,461 (28.7%)	26.7	0.304 (0.253, 0.365)	0.000	17.1	0.371 (0.29, 0.474)	0.000
Social insurance scheme	421 (8.3%)	30.4	0.69 (0.534, 0.892)	0.005	17.5	0.676 (0.481, 0.95)	0.024
Private health insurance	331 (6.5%)	50.6	1.157 (0.882, 1.517)	0.292	29.6	1.024 (0.722, 1.453)	0.892
Philanthropist	317 (6.2%)	20.8	0.157 (0.113, 0.217)	0.000	11.2	0.129 (0.074, 0.226)	0.000
*Type of hospital*							
Public	2,236 (43.9%)	18.6	Reference		9.2	Reference	
Semi-private	1,630 (32%)	39.9	2.980 (2.503, 3.547)	0.000	24.4	3.297 (2.595, 4.19)	0.000
Private	1,229 (24.1%)	36.7	2.188 (1.816, 2.636)	0.000	25.9	3.025 (2.343, 3.904)	0.000
*Duration of stay (in days)*							
1	592 (11.6%)	40.5	1.037 (1.031, 1.044)	0.000	24.1	1.043 (1.034, 1.052)	0.000
2	477 (9.4%)	18.0			10.2		
3	566 (11.1%)	19.9			9.9		
4	584 (11.5%)	18.0			7.1		
5	602 (11.8%)	21.8			11.2		
>5	2,274 (44.6%)	37.0			23.6		
Total	5,095	29.8			17.2		

a State government sponsored category includes patients enrolled in AB-PMJAY and other state health insurance schemes.

### Determinants of catastrophic health expenditure (CHE)

#### Outpatient treatment

The odds of incurring CHE were 10.9 times higher among poorest patients [AOR: 10.959 (95% CI: 7.636–15.728)] as compared to richest patients. Lower odds of CHE were observed among patients supported by philanthropist/charitable trusts [AOR: 0.258 (95%CI: 0.17–0.391)] and enrolled under state sponsored schemes [AOR: 0.5 (95%CI: 0.393–0.635)] and AB-PMJAY [AOR: 0.631 (95%CI: 0.429–0.93)]. There was lesser odds of incurring CHE among patients on hormone therapy [AOR: 0.326 (95%CI: 0.202–0.523), *p* value <0.001] as compared to chemotherapy. The odds of CHE were significantly higher for stage IV cancer [AOR: 1.806 (95%CI: 1.235–2.639)] as compared to stage I. Patients with ongoing response to treatment had a 46.2% higher likelihood of experiencing CHE [AOR: 1.462 (95%CI: 1.112–1.922), p value = 0.007] than those in the progression-free survival stage. Variables such as age, gender, level of education, marital status, type of cancer, cancer stage, type of treatment (except hormone therapy), line of treatment, and adverse effects of treatment did not significantly impact the odds of experiencing CHE due to outpatient cancer treatment (Table 2).

#### Hospitalization

The likelihood of experiencing CHE due to hospitalization was 7.4 times higher among the poorest [AOR: 7.414 (95%CI: 5.826–9.435), *p* < 0.05] as compared to richest income groups. The odds of incurring CHE were lower among patients covered under publically financed health insurance schemes namely AB PM-JAY [AOR: 0.426 (95%CI: 0.33–0.548)], state government sponsored schemes [AOR: 0.304 (95%CI: 0.253–0.365)], social insurance scheme [AOR: 0.69 (95%CI: 0.534–0.892)], and those supported by philanthropists/NGOs/trusts [AOR: 0.157 (95%CI: 0.113–0.217)] respectively. However, the odds of CHE were found to be approximately 15.7% higher for patients enrolled under private health insurance [AOR: 1.157 (95%CI: 0.882–1.517), *p* value = 0.292]. Hospitalization in private facilities [AOR: 2.188 (95%CI: 1.816–2.636)] and a longer duration of hospital stay [AOR: 1.037 (95%CI: 1.031–1.044)] were significantly associated with higher odds of CHE (Table 3).

### Impoverishment due to cancer treatment

**Figure 5:**
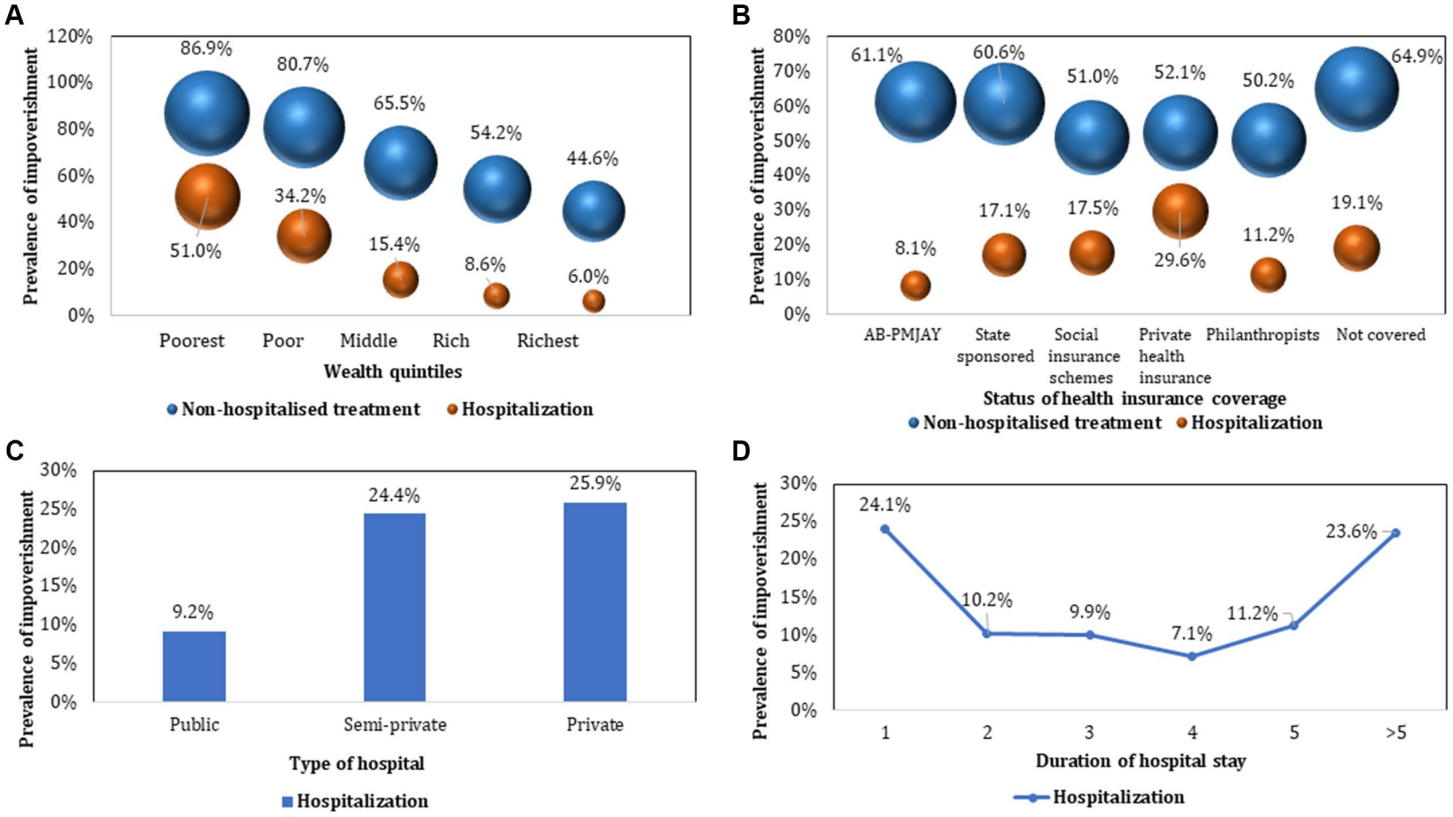
Prevalence of impoverishment due to cancer treatment.

The prevalence of impoverishment declined with increase in the level of income, from poorest to richest income groups for both hospitalisation (51% among poor versus 6% among rich) and outpatient treatment (86.9% among poor versus 44.6% among rich). Higher impoverishment rates were found among patients who sought hospitalization in private facilities (25.9%). Lowest impoverishment rates (8.1%) were observed among hospitalized patients enrolled under AB PM-JAY (Figure 5). The prevalence of impoverishment among different subgroups (stratified by socio-demographic and clinical characteristics) of cancer patients has been summarized in Table 3.

### Determinants of impoverishment due to cancer treatment

#### Outpatient treatment

The risk of incurring impoverished expenditures for outpatient treatment was 46.7 times higher among poorest income groups [AOR: 46.78 (95%CI: 21.101–103.71)] as compared to richest income groups. As compared to patients with no health insurance coverage, the likelihood of impoverishment was 40.3%, 49.7%, 60% and 56.6% lower among those covered under AB PM-JAY, state government sponsored, social insurance scheme, and patient support groups (philanthropists/NGOs) respectively (*p* < 0.05). The odds of impoverishment were lower among patients who received hormone therapy [AOR: 0.412 (95%CI: 0.239–0.709)] as compared to those on chemotherapy. Variables such as age, gender, marital status, level of education, type of cancer, stage of cancer, type of treatment (other than hormone therapy), line of treatment, and adverse effects of treatment had no significant effect on the odds of impoverishment (Table 2).

#### Hospitalization

The odds of impoverishment due to hospitalization were found 28.4 times higher among poorest [AOR: 28.432 (95%CI: 18.641–43.367)] as compared to the richest income groups. The odds of impoverishment for patients covered under health insurance schemes were lower among patients covered under AB PM-JAY [AOR: 0.395 (95%CI: 0.277–0.565)], state government sponsored schemes[AOR: 0.371 (95%CI: 0.29–0.474)], social insurance scheme [AOR: 0.676 (95%CI: 0.481–0.95)], and those supported by philanthropists/NGOs/trusts [AOR: 0.129 (95%CI: 0.074–0.226)] respectively. However, the odds of impoverishment were found to be approximately 2.4% (AOR: 1.024, *p* = 0.892) higher for patients having private health insurance. The patients hospitalised in private health facilities were nearly 3 times more likely to experience impoverishment (AOR: 3.025, *p* < 0.001) than those admitted in public hospitals. Similarly, likelihood of impoverishment was found to increase with every one unit increase in duration of hospital stay (AOR: 1.043, *p* < 0.001) (Table 3).

#### Indirect OOPE on cancer treatment

The total estimated indirect cost borne by a patient and caregiver per month due to outpatient treatment was INR 46,868 (1,164). Indirect cost borne by a patient and a caregiver per episode of hospitalization was computed as INR 25,173 (1,082).

The extent of CHE and impoverishment due to outpatient treatment increased from 83% (direct costs) to 99.7% (direct+ indirect costs) and, 63.9% (direct costs) to 97.1% after considering both direct and indirect costs borne by the patient and caregivers, respectively. In case of hospitalization, the extent of CHE increased from 23.6% (direct cost) to 59.4% (direct+ indirect costs) and impoverishment increased from 14.1% (direct cost) to 27% on considering both direct and indirect cost of cancer treatment.

### Impact of publically financed health insurance scheme

The prevalence of CHE due to hospitalization was lower among those covered under AB PM-JAY (16.1%) which was statistically significant (*p* < 0.001), as compared to those who were not covered by any health insurance (34.4%). Similarly, lower impoverishment rates (8.1%) were observed among hospitalized patients enrolled under AB PM-JAY.

The odds of incurring CHE due to hospitalization were lower among patients covered under publically financed health insurance schemes namely AB PM-JAY [AOR: 0.426 (95%CI: 0.33–0.548)], state government sponsored schemes [AOR: 0.304 (95%CI: 0.253–0.365)], social insurance scheme [AOR: 0.69 (95%CI: 0.534–0.892)], and those supported by philanthropists/NGOs/trusts [AOR: 0.157 (95%CI: 0.113–0.217)] respectively. However, the odds of CHE were found to be approximately 15.7% higher for patients enrolled under private health insurance [AOR: 1.157 (95%CI: 0.882–1.517), *p* value = 0.292].

Further, the odds of impoverishment for patients covered under health insurance schemes were lower among patients covered under AB PM-JAY [AOR: 0.395 (95%CI: 0.277–0.565)], state government sponsored schemes[AOR: 0.371 (95%CI: 0.29–0.474)], social insurance scheme [AOR: 0.676 (95%CI: 0.481–0.95)], and those supported by philanthropists/NGOs/trusts [AOR: 0.129 (95%CI: 0.074–0.226)] respectively. However, the odds of impoverishment were found to be approximately 2.4% (AOR: 1.024, *p* = 0.892) higher for patients having private health insurance.

In case of outpatient treatment, lower odds of CHE were observed among patients supported by philanthropist/charitable trusts [AOR: 0.258 (95%CI: 0.17–0.391)] and enrolled under state sponsored schemes [AOR: 0.5 (95%CI: 0.393–0.635)] and AB-PMJAY [AOR: 0.631 (95%CI: 0.429–0.93)] as compared to those who were not covered under any financial benefit scheme. The likelihood of impoverishment due to outpatient treatment was 40.3%, 49.7%, 60% and 56.6% lower among those covered under AB PM-JAY, state government sponsored, social insurance scheme, and patient support groups (philanthropists/NGOs) respectively (*p* < 0.05), as compared to patients with no health insurance coverage.

## Discussion

According to the recent National Health Accounts for India, about 49% of total health expenditure is paid entirely out-of-pocket (37). Despite the introduction of various publicly financed health insurance schemes, evidence on their impact on reducing reliance on OOPE incurred on cancer treatment especially since introduction of AB PM-JAY is scarce. We undertook this study to present a comprehensive picture of the economic burden of cancer from patients’ perspective. Our study fills the aforementioned gaps in the existing literature by collecting primary data from 9,897 cancer patients recruited across six states of the country.

### Overall summary of findings

Our study leads to four important findings. Firstly, the high economic burden for cancer treatment is due to outpatient care, rather than hospitalization, which has important bearing for the existing publically financed health insurance schemes which do not cover outpatient cancer care. Secondly, cancer care delivered in public sector leads to greater financial risk protection as compared to private sector which is an important argument for strengthening public sector for delivering cancer care. Thirdly, the publically financed insurance provides significantly higher financial risk protection. As a result, the benefit packages should be rationally expanded to increase the population coverage and include more cost-effective services. This will enable health maximization, provider higher financial risk protection and leads to universal health coverage. Together, with the first key finding, an important recommendation of the study is to design intervention to provide financial risk protection against outpatient treatment. An important determinant of OOPE for outpatient care is diagnostics (36.4%). Digital technological solutions may have an important role to play here. The Government of India recently launched a digital voucher to provide social assistance on subsidy. This digital voucher-E-RUPI (38), can be used to pay for expensive diagnostic and staging investigations once a patient has been confirmed for cancer following a histopathology. A pilot for such use case of E-RUPI has been planned by the AB PM-JAY. With more laboratory and diagnostic centres becoming part of the digital health ecosystem as part of Ayushman Bharat Digital Mission (ABDM), this can become even more effective. Further, expansion of E-RUPI can be used to pay for medicines in outpatient settings, which have been shown to constitute 27.8% of total OOPE. The fourth important implication of our study is generation of unit OOPE estimates, stratified by various clinical characteristics. These can be used along with health system cost database (39), to undertake oncology specific health technology assessments (40–42).

### Comparison with previously published literature

Globally, several studies have attempted to estimate the economic burden of cancer. However, a recent systematic review from India found only 22 studies reporting financial hardship among cancer patients (7, 14, 16–19, 43, 44). Most of these studies were single-centric with sample as small as 11, making the findings not generalizable for the country. Among the included studies, only 5 studies measured CHE and 11 studies assessed distress financing among cancer patients in India (7, 14, 16–19, 44). The estimated reported in these studies on OOPE, CHE, and impoverishment varied significantly. The OOPE incurred on cancer treatment ranged from ₹ 34,816 on head & neck, breast, and cervical cancer to ₹ 3,35,800 on palliative care (17, 20). This wide range of OOPE is due to heterogeneity within the studies owing to difference in study settings (single or multi-centric studies in one or more states of India), varied sample size ranging from 50 to 3,012, non-representative nature of included studies (varying expenditures on different cancers), type of OOPE reported (medical or non-medical or both), category of health services covered (outpatient or inpatient or both), and so on (16–19, 44). A thorough review of these studies revealed high heterogeneity in the methodological robustness and outcomes estimated (16–19, 44).

The review also reported that the proportion of cancer patients facing CHE ranged from 34% to 84% (8, 14, 17, 19, 21). High variability of these results observed are due to different thresholds, and proxies used for indicating capacity to pay (CTP) and computing CHE and impoverishment. While most studies used non-food consumption expenditure as proxy for CTP (14, 17, 21), other studies have used total household consumption expenditure (8) or annual household income (19) to determine the extent of CHE among cancer patients. It should also be noted that these studies used different cut-offs (10% and 40%) of CTP for reporting CHE. A few studies have also conducted sensitivity analysis to determine CHE at different cut-off levels, such as 20%, 30%, 40%, and 50% (8, 14, 16). It is worthwhile to mention that we have followed the standard methodological approach recommended by World Health Organization (WHO) using non-subsistence consumption expenditure as an indicator of CTP (40% threshold), for computation of CHE (35). This approach is superior to the older approaches used for estimation of CHE wherein CHE was defined as expenditure that exceeded a certain proportion of the income and total expenditure of the household in a certain period of time (45–47). However, the use of subsistence spending methods is more pro-equity. This is justified in view of the fact that the poor spend a disproportionately greater share of their overall income on subsistence expenditure, leaving very little to fend off other expenditures including health care spending. As per ICE 360 Survey 2014 by Peoples’ research on India’s Consumer Economy-PRICE (48), the poorest 20% of the income population spends nearly 67.7% of their total consumption expenditure on basic subsistence needs, as compared to 45.2% in the case of the richest 20% population. This implies that the financial hardship of a similar level of OOP spending on health is likely to be higher for a poor household, even more than what is simply explained by differences in the overall levels of income (or consumption expenditure) alone.

Moreover, given the kind of informal economy that prevails in India, there is under reporting of income. Hence, the true impact of economic burden cannot be assessed without reliable income data. In addition to this, poverty line is also arbitrarily defined and there are a lot of ongoing debates on defining the poverty line. Literature on deprivation and economic hardship is also now pointing towards multidimensional criteria for defining poverty rather than a single cut-off value. Further, there are practical difficulties in collecting a reliable income data which is widely acknowledged (49, 50). Hence, it is more appropriate to define CHE as a certain share of non-food consumption expenditure (household consumption expenditure minus subsistence expenditure, refers to ability to pay) as a measure to compute CHE and impoverishment. We have used a threshold of 40% of ability to pay, which has also been proposed for the definition of catastrophic health expenditure in a study of 59 countries in 2003 (34) and has also been suggested by WHO (28).

The determinants of CHE observed in our study are in line with those reported in previous studies (14, 16, 19, 21). The odds of CHE were found to be higher among poorer income groups as compared to richer income groups, which is consistent with our findings (14, 16, 21). This can be attributed to several factors which determine the extent of financial toxicity among poorest quintiles. Firstly, the income for the poor patients is significantly lower, given the large differentials of income inequality. Secondly, the ability to pay for health care costs, after accounting for basic subsistence expenditure is even lower for the poor patients who spend a significantly larger proportion of their income on consumption of basic needs such as food, leaving little to meet other needs such as healthcare expenditure (48). As a result, the proportion of the poor who face CHE is significantly higher than their richer counterparts. Further, these estimates also corroborates with findings of another study by Akhtar et al. (51) wherein the poorest 20% have been reported to have a prevalence of 77.5% CHE due to hospitalization as compared to 63.1% among richest 20% population.

Furthermore, patients without any health insurance had higher odds of experiencing CHE as compared to those covered under some health insurance scheme (19). A systematic review of impact evaluations of publically financed health insurance schemes in India showed no significant impact on OOPE and financial risk protection (52). Several subsequent studies also showed similar findings (53, 54). However, majority of these studies evaluated either pre-PM-JAY schemes or AB PM-JAY was in early phase. Our study provides positive effect of PM-JAY on OOPE and financial risk protection for cancer patients. Thirdly, stage II & above cancers (as compared to stage 1 cancer) and utilization of health services in private hospitals (in comparison to public hospitals) were found to be associated with higher odds of CHE (21). Late presentation of the disease and the need for more intensive treatment at later stages put cancer patients at a higher risk of financial catastrophe. Furthermore, very few studies have reported estimates on impoverishment; 31.4% due to hospitalization and 15.5% due to outpatient treatment (8). However, these estimates are slightly different from our study estimates. This variation can be attributed to different methodological approaches used to estimate impoverishment. We have used subsistence spending as poverty line in our analysis based on methodology given by Xu et al. (34), however other studies have used cut offs (separately for rural and urban) given by Tendulkar committee, Rangarajan committee etc (55).

The high impoverishment estimated in our study on account of expenditure for outpatient care can be explained by the following factors. Firstly, the cancer patients continue to incur a significantly high OOP expenditure on outpatient care, usually on account of chemotherapy as well as diagnostics for routine monitoring and supportive care. Per patient annual outpatient expenditure is 4.8 times that of the annual inpatient OOP expenditure. The repetitive nature of multiple outpatient care episodes also contributes to this high OOP expenditure, as compared to relatively low frequency of inpatient care. Our study estimates are consistent with findings of another study by Thomas et al. (56), assessing the impact of illness on hardship financing using National Sample survey Organization (NSSO) data from 72nd (2014) and 75th (2018) rounds. The marginal effects model used in the study reported that the probability of hardship financing by outpatient cases increased by 1.3% (*p* < 0.01, highly statistically significant) in the year 2018, in contrast to inpatient cases wherein it got decreased by 7.5% (*p* < 0.01) (56).

Secondly, majority of the publicly financed health insurance schemes include only inpatient care in its health benefits package, leaving outpatient care out of the ambit. Thirdly, even for the inpatient care which is covered, the financial protection starts to tick in once the diagnosis is established, which implies that the initial diagnostics and staging in case of probable cancer cases is paid out-of-pocket by the patients. Our study findings also suggests that diagnostics account for 36.4% of total OOPE incurred on non-hospitalized cancer treatment followed by medicines having a share of 27.8%.

### Policy implications

The study findings provide important policy implications. Firstly, the health benefits package of Ayushman Bharat PM-JAY should prioritise the expansion of cancer packages, by including the cost-effective treatments which may be delivered in outpatient care. Secondly, the digital payment systems should be used to finance the cost of diagnostic services availed for staging of cancer patients before the treatment begins.

### Strengths

We would like to mention several methodological strengths of our study. Firstly, this is the first study to have estimated OOPE, catastrophic health expenditure and impoverishment due to both outpatient and hospitalized cancer treatment on such a large sample of 12,148 cancer patients. Secondly, the data were collected from patients recruited at seven health care facilities across six states of the country. Two of the selected hospitals in our sample, are among the top 10 hospitals in terms of cancer treatment claims as part of the largest insurance scheme in India-AB PM-JAY (57). Thirdly, our sample population included cancer patients from all age groups, socio-economic categories, and with any type of cancer (solid and haematological), thus making it more representative and generalizable. Fourthly, our study sample is sufficient to provide valid estimates of OOPE and CHE for top 11 and 17 cancers, respectively, in India, with a 10% margin of error and 95% confidence intervals. Fourthly, the study used actual data on number of outpatient visits per month and episodes of hospitalization per year to determine annual OOPE which demonstrated the true impact of OOPE. We would like to highlight that besides the large sample size and external validity of estimates, the other contribution is that we provide site-specific estimates of OOP expenditure along with determinants of CHE and impoverishment (Supplementary Table S6, Supplementary Appendix S5).

Furthermore, our study is the first study which estimates the extent of OOP expenditure in the post-PMJAY, and measuring the impact of the PM-JAY on reducing financial hardship. In a way, our study is the first of its kind which shows a positive effect of a government funded health insurance scheme on the OOP expenditure and catastrophic spending for healthcare in India.

### Limitations

Our study also has certain limitations. Firstly, the OOPE estimates for outpatient treatment are applicable to public and semi-private hospitals in India. Although, three out of seven selected health care facilities cater services to four categories of patients (general, private, referrals, and preventive oncology). Therefore, inpatients charges for private category represents private sector. Secondly, some part of the data collection occurred during the Covid-19 pandemic, which could have negatively impacted the non-food consumption expenditure among cancer patients and their families due to loss of earnings, further increasing the financial risk due to cancer treatment.

## Conclusion

There is high economic burden on patients and their families for cancer treatment. Our findings emphasize the need for urgent strategies to mitigate financial toxicity among cancer patients in India, especially in the most deprived sections of society. There is also a need to have a broader consultation group including multiple stakeholders such as healthcare providers, government agencies, insurance companies, and patient advocacy groups so as to make comprehensive strategies to address the issue of financial toxicity in cancer patients in India. The increase in coverage of PFHI schemes, rationalization of HBP’s, strengthening public health care facilities, creating prepayment mechanism like E-RUPI in the form of social insurance can potentially reduce the financial distress among cancer patients in India (38). The disaggregated estimates by site of cancer, stage, type of treatment and treatment response could be useful input for future health technology analyses to determine the cost-effective treatment strategies.

Future research should aim at undertaking a more longitudinal follow-up study to determine the non-health consequences of cancer. This should include the social consequences of health care expenditures on cancer, such as leading to drop out of children from school, lack of ability to carry out other social responsibilities, need to curtail other important spending etc. In addition, such a study could also lead to intergenerational impact of cancer expenditures. Secondly, the future research should also more rigorously examine the effectiveness of different measures of reducing the OOP spending on cancer. Thirdly, a more detailed examination of the distributional impact of OOP expenditures for cancer, as well as strategies to improve the access for the most needy and vulnerable needs to be undertaken. This is also in line with the global call for the cancer day (58).

## Data availability statement

The original contributions presented in the study are included in the article/Supplementary material, further inquiries can be directed to the corresponding author.

## Ethics statement

The study was reviewed and approved by the Institutional Ethics Committee of Post Graduate Institute of Medical Education and Research, India with reference number IEC-03/20202-1565 as it involved human participants. A written informed consent was obtained from all study participants.

## Author contributions

SP: conceptualization, funding acquisition, resources, and supervision. SP, JD, and AD: data curation and writing – original draft. SP, JD, and AG: formal analysis. SP and JD: investigation, methodology, project administration, and software. SP, JD, NG, AD, AK, PR, NM, LK, AS, PM, AG, KR, MK, and SG: validation, visualization, and writing – review and editing. All authors contributed to the article and approved the submitted version.

## Funding

The study was funded by the Department of Health Research, Ministry of Health and Family Welfare, Government of India vide grant number F.No.T.11011/02/2017-HR/3100291. However, the funder did not play a role in the design of the study; the collection, analysis, and interpretation of the data; the writing of the manuscript; and the decision to submit the manuscript for publication. We did not receive any funds for paying open access publication fees from our institutions, library or any other source.

## Conflict of interest

The authors declare that the research was conducted in the absence of any commercial or financial relationships that could be construed as a potential conflict of interest.

## Publisher’s note

All claims expressed in this article are solely those of the authors and do not necessarily represent those of their affiliated organizations, or those of the publisher, the editors and the reviewers. Any product that may be evaluated in this article, or claim that may be made by its manufacturer, is not guaranteed or endorsed by the publisher.

## Supplementary material

The Supplementary material for this article can be found online at: https://www.frontiersin.org/articles/10.3389/fpubh.2023.1065737/full#supplementary-material
