# Supplementary material for: Financial toxicity of cancer treatment in India: towards closing the cancer care gap
Source: Front Public Health. 2023 Jun 19;11:1065737. doi: 10.3389/fpubh.2023.1065737 (PMC10316647; doi:10.3389/fpubh.2023.1065737)
Supplement: Supplementary file 1 [file Table_1.DOCX]

Supplementary appendix

Table of Contents

[Supplementary Appendix: S1 2](#_Toc130857838)

[Table 1: Summary of data collection at selected sites 2](#_Toc130857839)

[Case Definitions 3](#_Toc130857840)

[Supplementary Appendix: S2 6](#_Toc130857841)

[Data collection tool for direct expenditure due to outpatient treatment 6](#_Toc130857842)

[Supplementary Appendix: S3 10](#_Toc130857844)

[Data Collection Tool for direct expenditure due to hospitalization 10](#_Toc130857845)

[Supplementary Appendix: S4 15](#_Toc130857848)

[Supplementary Appendix: S5 18](#_Toc130857849)

[Mean direct OOPE on outpatient treatment and its association with clinical characteristics of cancer patients 21](#_Toc130857851)

[Mean direct OOPE due to hospitalization and its association with socio-demographic and clinical characteristics of cancer patients 23](#_Toc130857853)

[Determinants of out-of-pocket expenditure due to outpatient and hospitalized treatment 25](#_Toc130857854)

[Health care burden due to cancer, stratified according to primary cancer site 27](#_Toc130857857)

# Supplementary Appendix: S1

Table 1: Summary of data collection at selected sites

| Duration of data collection | Name of the Centre | Sample size |
| --- | --- | --- |
|  |  | **OPD** |
| 05 /10 /2020 to 24/03/2022 | **GMCH-32, Chandigarh** | 2395 |
| 06/10/2020 to 24/03/2022 | **BBCI, Assam** | 2073 |
| 8/10/2020 to 27/08/2021 | **PGIMER, Chandigarh** | 931 |
| 07/10/2020 to 15/11/2021 | **AIIMS, New Delhi** | 1243 |
| 02/12/2020 to 24/03/2022 | **Adyar Cancer Centre, Chennai** | 1330 |
| 05/01/2021 to 31/12/2021 | **CMC, Vellore** | 935 |
| 07/10/2021 to 24/03/2022 | **Tata Memorial Hospital, Mumbai** | 880 |
| Total | **All sites** | 9787 |

## Case Definitions

1. **NEWLY DIAGNOSED CASE:**

Newly diagnosed case is defined as a patient who has received a diagnosis of malignancy (histopathology proven) in ≤ 45 days prior to study inclusion and who has not received any cancer directed treatment plan.

Data collection plan:

**Retrospective data collection**

Patient inclusion in to the study (d0)

-30 days

1. **ON TREATMENT CASE:**

On treatment case is defined as those who are receiving any form of cancer directed treatment at the first time of study inclusion or within prior to the 1 year of study inclusion.

Data collection plan:

1. Retrospective data collection: If the last visit of the patient is ≤30 days, retrospective data on out-of-pocket expenditure incurred for outpatient treatment in the past 1 month or since last visit (whichever is less) will be collected along with assessment of health related quality of life (HRQOL).

**Retrospective data collection**

Patient inclusion in to the study (d0)

-30 days

1. **FOLLOW-UP CASE:**

Follow-up case are those patients who have been diagnosed more than one year prior to study inclusion and are not receiving a planned cancer treatment at the time of study inclusion.

Data collection plan:

1. Prospective data collection: If the last visit of the patient is ≥30 days, prospective data will be collected after + 15 ± 2 days

**Prospective data collection**

+ 15 ± 2 days

Patient inclusion in to the study (d0)

1. Retrospective data collection:
2. If the last visit of the patient is ≤ 30 days, retrospective data on out-of-pocket expenditure incurred for outpatient treatment in the past 1 month or since the last visit (whichever is less) will be collected along with the assessment of HRQOL.

**Retrospective data collection**

Patient inclusion in to the study (d0)

-30 days

1. All the out-of-pocket expenditures incurred for the utilization of inpatient services due to cancer in the past 12 months prior to the inclusion into the study. This is applicable to all the three types of patients-newly diagnosed, on-treatment cases and follow-up cases.

**Retrospective data collection**

Patient inclusion in to the study (d0)

-12 months

**Inpatients/hospitalised cases:**

In-patients are defined as those patients who have been **hospitalized overnight** and they will be categorised as per their definition of the type of cases as newly diagnosed, on-treatment and follow-up cases.

*** Details of the case types and the elements of data collection:**

| **S. No.** | **Case type** | **Cancer diagnosis** | **Cancer treatment** | **Type of data collection** | **Time period for data collection** | **Elements of data collection** |
| --- | --- | --- | --- | --- | --- | --- |
| **Outpatient cases** | | | | | | |
| 1 | Newly diagnosed | ≤ 45 days prior to study inclusion | Should not have happened | Retrospective data | -30 days from study inclusion | 1 – 7 |
| 2 | On-treatment | Any time prior to study inclusion | Yes; last cancer treatment of any type, within 12 months of study inclusion | a) Prospective data (applicable to patients with prior visit > 30 days) | + 15 ± 2 days from study inclusion | 1 – 7 |
|  |  |  |  | b) Retrospective component (applicable to patients with prior visit ≤ 30 days) | -30 days from study inclusion | 1 – 7 |
|  |  |  |  | c) Retrospective data to capture information related to hospitalization (applicable to all patients on treatment) | -12 months from study inclusion | 1 – 8 |
| 3 | Follow-up treatment | Any time prior to study inclusion | Completed cancer treatment more than 12 months of study inclusion and are visiting hospital for follow-up | a) Prospective data (applicable to patients with prior visit > 30 days) | + 15 ± 2 days from study inclusion | 1 – 7 |
|  |  |  |  | b) Retrospective component (applicable to patients with last visit ≤ 30 days) | -30 days from study inclusion | 1 – 7 |
|  |  |  |  | c) Retrospective data to capture information related to hospitalization (applicable to all patients on treatment) | -12 months from study inclusion | 1 – 8 |
| 4 | Inpatients (categorized into as per their definition of the type of cases as newly diagnosed, on-treatment & follow-up cases) | Patients will be interviewed daily till discharge to collect information on daily expenses incurred on hospitalization including inpatient stay in cancer ward/HDU/ICU/surgical procedure in inpatient setting etc. during last one year. However, rest of the information such as socio-demographic characteristics, consumption expenditure, clinical information and HRQOL will be recorded on the day of recruitment | | | | |

***Elements of data collection:*** *Consultation charges-1,* *Lab investigations/Diagnostics-2,* *Drug costs-3, Day care charges-4, Radiation charges-5, Transportation charges-6, Health related quality of life-7, Hospitalization charges-8*

# Supplementary Appendix: S2

## Data collection tool for direct expenditure due to outpatient treatment

**SECTION-A: GENERAL INFORMATION**

CR No.…………………. Patient ID: ___ ___ /____ /___ ___ ___ ___

Name of the Department…………… Dept. Registration No (if applicable)………………….……….

Name of the clinic (if applicable) ………………. Date of Interview ----/----/----

1. Name of Patient …………………………………………………………………...………….......
2. Name of care-giver (if patient is not the respondent)……………………………………………..
3. Contact No. of respondent

Home______________________Mobile-1__________________Mobile-2_________________

1. Gender :

a) Male b) Female

1. Age of Patient in years months
2. Religion :

a) Hindu b) Muslim c) Sikh

d) Christian f) Others

1. Area of residence :
2. Urban b) Slum c) Rural
3. Educational status :

a) Illiterate b) Primary c) Middle

d) Matric e) Senior secondary f) Graduation

g) Post graduation

1. Marital Status :

a) Unmarried b) Married

c) Separated/Divorced d) Widow/Widower

1. Financial benefit scheme :

a) Ayushman Bharat PradhanMantari Jan ArogyaYojana (AB-PMJAY) b) Other centrally sponsored schemes b) State government sponsored c) Government/PSU as an employer d) Employer supported (other than govt./PSU) health protection e) Voluntary private insurance f) Philanthropists/NGO’s/trusts g) Others. Specify……..h) Not covered

1. Total number of family members……………..
2. Total number of family members aged >10 years……………..
3. Total number of family members aged <10 years……………..

**SECTION B: OUT OF POCKET EXPENDITURE**

| 1. **.How much was the expenditure incurred on outpatient treatment since the last visit (in ₹). [Fill this section after 15 days if last visit was more than a month ago** | | **B). In regard to the above expenditure incurred, what was the source of finance?** | |
| --- | --- | --- | --- |
| **Expenditure Head** | **Amount (in ₹)** | **Source** | **Amount (in ₹)** |
| Travelling cost |  | Salary/Savings |  |
| Medicines |  | Selling of assets |  |
| Lab tests/ Diagnostics |  | Borrowed from relatives/friends without interest |  |
| User fees/Hospital charges (File charges) |  | Borrowed with interest |  |
| Informal payment |  | Health insurance |  |
| Boarding/Lodging |  | Any other (specify) |  |
| Food |  |  |  |
| Other |  |  |  |
| Total |  |  |  |

1. **Please provide information related to any hospitalization occurred due to cancer during last one year**

| Hospital admission | Type of hospital (Public/Private) | Reason of hospitalization | Number of days of admission | Total OOPE for an episode of hospitalization |
| --- | --- | --- | --- | --- |
| 1 |  |  |  |  |
| 2 |  |  |  |  |
| 3 |  |  |  |  |
| 4 |  |  |  |  |
| 5 |  |  |  |  |
| 6 |  |  |  |  |

# SECTION C: EQ-5D-5L tool for estimation of health-related quality of life. Under each heading, please tick the ONE box that best describes your health TODAY

**MOBILITY**

I have no problems in walking about ❑

I have slight problems in walking about ❑

I have moderate problems in walking about ❑

I have severe problems in walking about ❑

I am unable to walk about ❑

**SELF-CARE**

I have no problems in bathing or dressing myself ❑

I have slight problems in bathing or dressing myself ❑

I have moderate problems in bathing or dressing myself ❑

I have severe problems in bathing or dressing myself ❑

I am unable to bathe or dress myself ❑

**USUAL ACTIVITIES** (e.g. work, study, housework, ❑

family or leisure activities)

I have no problems doing my usual activities ❑

I have slight problems doing my usual activities ❑

I have moderate problems doing my usual activities ❑

I have severe problems doing my usual activities ❑

I am unable to do my usual activities ❑

**PAIN / DISCOMFORT**

I have no pain or discomfort ❑

I have slight pain or discomfort ❑

I have moderate pain or discomfort ❑

I have severe pain or discomfort ❑

I have extreme pain or discomfort ❑

**ANXIETY / DEPRESSION**

I am not anxious or depressed ❑

I am slightly anxious or depressed ❑

I am moderately anxious or depressed ❑

I am severely anxious or depressed ❑

I am extremely anxious or depressed ❑

We would like to know how good or bad your health is TODAY.

This scale is numbered from 0 to 100.

100 means the best health you can imagine.
0 means the worst health you can imagine.

Mark an X on the scale to indicate how your health is TODAY.

**SECTION-D CONSUMPTION EXPENDITURE**

| How much does your family spend per month on following items? | Expense | | |
| --- | --- | --- | --- |
|  | 7 days | 30 days | 365 days |
| 1. Food purchased/home production: ration (Cereals, pulses, edible oil, bread etc.), Fruits and vegetables, Milk, Milk products, Beverages etc. |  |  |  |
| 1. Education (Books, newspaper, fees) |  |  |  |
| 1. Health |  |  |  |
| 1. Bills (Electricity, telephone, water, Equated monthly installment –EMI etc.) |  |  |  |
| 1. Conveyance, fuel |  |  |  |
| 1. Rents |  |  |  |
| 1. Clothing, Footwear, bedding, curtains etc. |  |  |  |
| 1. Entertainment (Cable, cinema, sports, recreation & hobbies) |  |  |  |
| 1. Personal effects (Watch, mobile phone, spectacles, toiletries, jewelry) |  |  |  |
| 1. Consumer services (Domestic help, cook, sweeper, barber, tailor, priest, beautician) |  |  |  |
| 1. Pan, Tobacco, alcohol or any other intoxicants |  |  |  |
| 1. Miscellaneous (household appliances, furniture, crockery, animals, or any family function) |  |  |  |

**SECTION E: CLINICAL PROFILE OF CANCER PATIENTS**

|  | Patient ID: | ___ ___ /____ /___ ___ ___ ___ |
| --- | --- | --- |
|  | Date of Diagnosis |  |
|  | Start Date of treatment |  |
|  | Basis of Diagnosis  (multiple select) | - Clinical - Radiology (X-ray, USG, MRI,CT, PET) - Endoscopy - Histology - Cytology (FNAC/fluid cytology) - Peripheral blood immunophenotyping - Bone Marrow examination - Others, specify………………… - Unknown/No information |
|  | Primary Site |  |
|  | TNM Classification |  |
|  | Stage (single select) | - In situ - Localization (T1) - Direct Extension (T2+) - Regional lymph node involvement (N+) - Direct extension with regional lymph node involvement(T+N+) - Distant metastasis(M+) - Unknown/No information - Not applicable |
|  | Site specific staging |  |
|  | Histology |  |
|  | Final Diagnosis ICD-O 3 Classification |  |
|  | Final Diagnosis ICD-10 Classification |  |
|  | Treatment obtained since last visit (multiple select) | - Surgery - Radiotherapy - Brachytherapy - Chemotherapy - Chemotherapy+ Radiation - Surgery +Radiotherapy - Surgery +Chemotherapy - Surgery + Chemotherapy +Radiotherapy - Chemo-radiotherapy - Palliative Care - Unknown/No information - Others, specify………………. |
|  | Completion of Treatment (single Select) | - Complete - Ongoing - Not started yet - Refused further treatment - Unknown/No information |
|  | Adverse effect of treatment (select multiple options) | - Nausea - Vomiting - Diarrhoea - Mucositis - Hair loss - Fatigue - Weight loss - Anemia - Neutropenia - Febrile Neutropenia - Infections, not related to neutropenia - Deep Vein Thrombosis - Cardiac Complication - Second malignancy - Any other, please specify |
|  | Response to treatment | - Complete response - Very good partial response (applicable only for Multiple Myeloma) - Ongoing response - Partial response - Stable disease - Progressive disease - Minimal residual disease status negative (optional) |
|  | Line of treatment | - First line - Second line - Third line - Fourth line - If any other, specify………………….. |
|  | If on Chemotherapy, then ***current*** regimen/medication |  |

**Reason of hospitalization: Chemotherapy (1) /radiotherapy (2) /adverse event(3) /surgery (4)/others 5) , specify………………..**

# Supplementary Appendix: S3

## Data Collection Tool for direct expenditure due to hospitalization

**SECTION-A: GENERAL INFORMATION**

CR No.…………………. Patient ID: ___ ___ /____ /___ ___ ___ ___

Name of the Dept ………......... Dept. Registration No (if applicable)…………….....

Name of the Clinic (if applicable) ………… Date of Interview ----/----/----

1. Name of the Patient …………………………………………………………………...………….......
2. Name of the care-giver (if patient is not the respondent)……………………………………………..
3. Contact No. of respondent

Home______________________Mobile-1__________________Mobile-2_________________

1. Gender:

a) Male b) Female

1. Age of Patient in years months
2. Religion:

a) Hindu b) Muslim c) Sikh

d) Christian e) Others

1. Area of residence:
2. Urban b) Slum c) Rural
3. Educational status:

a) Illiterate b) Primary c) Middle

d) Matric e) Senior secondary f) Graduation

g) Post graduation

1. Marital Status:

a) Unmarried b) Married

c) Separated/Divorced d) Widow/Widower

1. Financial benefit scheme*: ……………………………
2. Total number of family members……………..
3. Total number of family members aged >10 years……………..
4. Total number of family members aged <10 years……………..

** 1-Ayushman Bharat Pradhan Mantari Jan ArogyaYojana (AB-PMJAY), 2-Other centrally sponsored schemes, 3- State government sponsored, 4- Government/PSU as an employer, 5-Employer supported (other than govt./PSU) health protection, 6-Voluntary private insurance, 7-Philanthropists/NGO’s/trusts, 8-Others, specify in Q.10, 9-Not covered*

**SECTION B: OUT OF POCKET EXPENDITURE**

1. **In regard to the expenditure incurred on hospitalization (See II), what was the source of finance*?**

| Source | **Amount in ₹** |
| --- | --- |
| Salary/Savings |  |
| Selling of assets |  |
| Borrowed from relatives/friends without interest |  |
| Borrowed with interest |  |
| Health insurance |  |
| Any other (specify) |  |

****Fill this section after filling Part II on next page***

1. **How much you spend on hospitalized care during last 24 hours (Day-wise)***

| **Expenditure Head** | **Day** | | | | | | | |  |
| --- | --- | --- | --- | --- | --- | --- | --- | --- | --- |
|  | 1 | 2 | 3 | 4 | 5 | 6 | 7 | 8 | 9 |
| Travelling cost |  |  |  |  |  |  |  |  |  |
| Medicines |  |  |  |  |  |  |  |  |  |
| Lab tests/ Diagnostics |  |  |  |  |  |  |  |  |  |
| Procedure/Surgery |  |  |  |  |  |  |  |  |  |
| User fees/Bed charges |  |  |  |  |  |  |  |  |  |
| Informal payment |  |  |  |  |  |  |  |  |  |
| Boarding/Lodging/Food |  |  |  |  |  |  |  |  |  |
| Others |  |  |  |  |  |  |  |  |  |
| Total |  |  |  |  |  |  |  |  |  |
| **Expenditure Head** | 10 | 11 | 12 | 13 | 14 | 15 | 16 | 17 | 18 |
| Travelling cost |  |  |  |  |  |  |  |  |  |
| Medicines |  |  |  |  |  |  |  |  |  |
| Lab tests/ Diagnostics |  |  |  |  |  |  |  |  |  |
| Procedure/Surgery |  |  |  |  |  |  |  |  |  |
| User fees/Bed charges |  |  |  |  |  |  |  |  |  |
| Informal payment |  |  |  |  |  |  |  |  |  |
| Boarding/Lodging/Food |  |  |  |  |  |  |  |  |  |
| Others |  |  |  |  |  |  |  |  |  |
| Total |  |  |  |  |  |  |  |  |  |
| **Expenditure Head** | 19 | 20 | 21 | 22 | 23 | 24 | 25 | 26 | 27 |
| Travelling cost |  |  |  |  |  |  |  |  |  |
| Medicines |  |  |  |  |  |  |  |  |  |
| Lab tests/ Diagnostics |  |  |  |  |  |  |  |  |  |
| Procedure/Surgery |  |  |  |  |  |  |  |  |  |
| User fees/Bed charges |  |  |  |  |  |  |  |  |  |
| Informal payment |  |  |  |  |  |  |  |  |  |
| Boarding/Lodging/Food |  |  |  |  |  |  |  |  |  |
| Others |  |  |  |  |  |  |  |  |  |
| Total |  |  |  |  |  |  |  |  |  |

**Fill this section for all days of admission till the patient is discharged*

# SECTION C: EQ-5D-5L TOOL FOR ESTIMATION OF HEALTH-RELATED QUALITY OF LIFE

#

# Under each heading, please tick the ONE box that best describes your health TODAY

We would like to know how good or bad your health is TODAY.

This scale is numbered from 0 to 100.

100 means the best health you can imagine.
0 means the worst health you can imagine.

Mark an X on the scale to indicate how your health is TODAY.

**MOBILITY**

I have no problems in walking about ❑

I have slight problems in walking about ❑

I have moderate problems in walking about ❑

I have severe problems in walking about ❑

I am unable to walk about ❑

**SELF-CARE**

I have no problems in bathing or dressing myself ❑

I have slight problems in bathing or dressing myself ❑

I have moderate problems in bathing or dressing myself ❑

I have severe problems in bathing or dressing myself ❑

I am unable to bathe or dress myself ❑

**USUAL ACTIVITIES** (e.g. work, study, housework, ❑

family or leisure activities)

I have no problems doing my usual activities ❑

I have slight problems doing my usual activities ❑

I have moderate problems doing my usual activities ❑

I have severe problems doing my usual activities ❑

I am unable to do my usual activities ❑

**PAIN / DISCOMFORT**

I have no pain or discomfort ❑

I have slight pain or discomfort ❑

I have moderate pain or discomfort ❑

I have severe pain or discomfort ❑

I have extreme pain or discomfort ❑

**ANXIETY / DEPRESSION**

I am not anxious or depressed ❑

I am slightly anxious or depressed ❑

I am moderately anxious or depressed ❑

I am severely anxious or depressed ❑

I am extremely anxious or depressed ❑

**SECTION-D CONSUMPTION EXPENDITURE**

| How much does your family spend on following items? | Expense | | |
| --- | --- | --- | --- |
|  | 7 days | 30 days | 365 days |
| 1. Food purchased/home production: ration (Cereals, pulses, edible oil, bread etc.), Fruits and vegetables, Milk, Milk products, Beverages etc. |  |  |  |
| 1. Education (Books, newspaper, fees) |  |  |  |
| 1. Health |  |  |  |
| 1. Bills (Electricity, telephone, water, EMI etc.) |  |  |  |
| 1. Conveyance, fuel |  |  |  |
| 1. Rents |  |  |  |
| 1. Entertainment (Cable, cinema, sports, recreation & hobbies) |  |  |  |
| 1. Consumer services (Domestic help, cook, sweeper, barber, tailor, priest, beautician) |  |  |  |
| 1. Pan, Tobacco, alcohol or any other intoxicants |  |  |  |
| 1. Clothing, Footwear, bedding, curtains etc. |  |  |  |
| 1. Personal items (Watch, mobile phone, spectacles, toiletries, jewelry) |  |  |  |
| 1. Miscellaneous (household appliances, furniture, crockery, animals, or any family function) |  |  |  |

**SECTION E: CLINICAL PROFILE OF CANCER PATIENTS**

|  | Patient ID: | ___ ___ /____ /___ ___ ___ ___ |
| --- | --- | --- |
|  | Date of Diagnosis |  |
|  | Start Date of treatment |  |
|  | Basis of Diagnosis  (multiple select) | - Clinical - Radiology (X-ray, USG, MRI,CT, PET) - Endoscopy - Histology - Cytology (FNAC/fluid cytology) - Peripheral blood immunophenotyping - Bone Marrow examination - Others, specify………………… - Unknown/No information |
|  | Primary Site |  |
|  | TNM Classification |  |
|  | Stage(single select) | - In situ - Localization (T1) - Direct Extension (T2+) - Regional lymph node involvement (N+) - Direct extension with regional lymph node involvement(T+N+) - Distant metastasis(M+) - Unknown/No information - Not applicable |
|  | Site specific staging |  |
|  | Histology |  |
|  | Final Diagnosis ICD-O 3  Classification |  |
|  | Final Diagnosis ICD-10  Classification |  |
|  | Treatment obtained since last visit (multiple select) | - Surgery - Radiotherapy - Brachytherapy - Chemotherapy - Chemotherapy+ Radiotherapy - Surgery +Radiotherapy - Surgery +Chemotherapy - Surgery + Chemotherapy +Radio - Chemo-radiotherapy - Palliative Care - Unknown/No information - Others, specify………………. |
|  | Completion of Treatment  (single Select) | - Complete - Ongoing - Not started yet - Refused further treatment - Unknown/No information |
|  | Adverse effect of treatment (select multiple options) | - Nausea - Vomiting - Diarrhoea - Mucositis - Hair loss - Fatigue - Weight loss - Anemia - Neutropenia - Febrile Neutropenia - Infections, not related to neutropenia - Deep Vein Thrombosis - Cardiac Complication - Second malignancy - If any other, specify…………….. |
|  | Response to treatment | - Complete response - Very good partial response - Ongoing response - Partial response - Stable disease - Progressive disease - Minimal residual disease status negative (optional) |
|  | Line of treatment | - First line - Second line - Third line - Fourth line - If any other, specify……………. |
|  | If on Chemotherapy, then ***current*** regimen/medication |  |

# Supplementary Appendix: S4

**Data Collection Tool for Indirect expenditure due to treatment**

***Patient Details:***

1. What would you have being doing otherwise if you were not taking treatment? (Multiple response allowed)

Time spent (in hours) on:

1 day 1 week 1 month

Household activities ____ ____ ____

Childcare ____ ____ ____

Professional work ____ ____ ____

Voluntary work ____ ____ ____

Leisure activities ____ ____ ____

Attending School/University ____ ____ ____

Seeking work ____ ____ ____

Social work ____ ____ ____

Physical workout ____ ____ ____

Other (specify) ____ ____ ____

1. Did other people take over and perform your usual household tasks during your hospital stay? If yes, fill the appropriate option, there can be more than one answer

Yes/No/NA Paid/Unpaid No. of hours

Household activities ____ ____ ____

Childcare ____ ____ ____

Professional work ____ ____ ____

Voluntary work ____ ____ ____

Leisure activities ____ ____ ____

Attending School/University ____ ____ ____

Seeking work ____ ____ ____

Social work ____ ____ ____

Physical workout ____ ____ ____

Other (specify) ____ ____ ____

**Caregivers:**

|  | Caregiver 1 | Caregiver 2 | Caregiver 3 |
| --- | --- | --- | --- |
| Relation with patient |  |  |  |
| Address |  |  |  |
| Contact No. |  |  |  |
| No. of visits (per day) |  |  |  |
| Total duration of Hospital stay (In hours) |  |  |  |
| Employment status (Yes/No) |  |  |  |
| Nature of employment (Give codes as mentioned in the end of tool) |  |  |  |
| Monthly Gross Income of Caregiver (In Rs) |  |  |  |
| **Time spent daily (hours) on:** |  |  |  |
| **Household activities** |  |  |  |
| Hours forgone due to care-giving |  |  |  |
| Alternative |  |  |  |
| No. of hours (alternative) |  |  |  |
| Payment to alternative paid worker (In Rs) |  |  |  |
| **Childcare** |  |  |  |
| Hours forgone due to care-giving |  |  |  |
| Alternative |  |  |  |
| No. of hours (alternative) |  |  |  |
| Payment to alternative paid worker (In Rs) |  |  |  |
| **Professional work** |  |  |  |
| Hours forgone due to care-giving |  |  |  |
| Alternative |  |  |  |
| No. of hours (alternative) |  |  |  |
| Payment to alternative paid worker (In Rs) |  |  |  |
| **Voluntary work** |  |  |  |
| Hours forgone due to care-giving |  |  |  |
| Alternative |  |  |  |
| No. of hours (alternative) |  |  |  |
| Payment to alternative paid worker (In Rs) |  |  |  |
| **Leisure activities** |  |  |  |
| Hours forgone due to care-giving |  |  |  |
| **Attending School/university** |  |  |  |
| Hours forgone due to care-giving |  |  |  |
| **Seeking work** |  |  |  |
| Hours forgone due to care-giving |  |  |  |
| **Social work** |  |  |  |
| Hours forgone due to care-giving |  |  |  |
| Alternative |  |  |  |
| No. of hours (alternative) |  |  |  |
| **Physical workout** |  |  |  |
| Hours forgone due to care-giving |  |  |  |
| **Other (specify)** |  |  |  |
| Hours forgone due to care-giving |  |  |  |
| Alternative |  |  |  |
| No. of hours (alternative) |  |  |  |

**Alternative Worker;* Yes (Paid) =1*,* Yes (Unpaid) =2*,* No=3*,* Not Applicable (NA) =4

*Employment Status;* Cultivator=1, Agricultural wage labourer=2, Non-agricultural wage labourer=3, Own account worker=4, Employer=5, Unpaid family worker=6, Regular salaried/Wage employee=7, Unemployed=8, Rentier/Pensioner/Other remittance recipient=9, Not able to work due to disability=10, Too old to work=11, Others=12

# Supplementary Appendix: S5

## Determinants of out-of-pocket expenditure: Outpatient and hospitalised treatment

A stratified analysis was done to determine the association between mean OOPE and socio-demographic characteristics of cancer patients (Table 1). The difference in OOPE estimates for outpatient treatment was found to be statistically significant (p<0.001) for various parameters, such as age, gender, level of education, wealth quintile and health insurance status. However, area of residence and marital status showed no significant association (p>0.05) with OOPE.

**Mean direct OOPE on outpatient treatment and its association with socio-demographic characteristics of cancer patients**

Mean OOPE incurred was found to be the highest among patients above 60 years of age [₹ 8,900 (293.6)] followed by those in the age group of 0-15 years [₹ 8,334 (648.3)], 45-60 years [₹ 7,977 (237.0)], 16-30 years [₹ 7,663 (411.3)], and 31-45 years [₹ 7,537 (287.6)]. OOPE was also found to be higher among males [₹ 8,907 (227.5)] as compared to females [₹ 7,444 (184.7). The OOPE was found to be increasing with increase in level of education [₹ 8,585 (293.2) for up to senior secondary education and ₹ 10,545 (542.8) for graduation and above]. However, OOPE incurred by patients with primary and middle level education [₹ 7,137 (205)] or no education [₹ 7,292 (229.5)] was comparable. Highest OOPE was incurred by patients belonging to richest income quintiles [₹ 12,260 (394.5)] followed by rich [₹ 9,307 (323.4)], middle [₹ 7,565 (319.3)], poor [₹ 6,301 (313.1)], and the poorest [₹ 4839 (198.2)] wealth quintiles. Patients who were not covered under any health insurance schemes incurred the highest OOPE [₹ 10,092 (278.5)]. Patients insured through philanthropists/NGOs incurred the lowest expenditure [₹ 4,164 (315)] followed by those who were enrolled in state government sponsored publically financed health insurance schemes [₹ 5840 (174.3)], AB-PMJAY [₹ 7,989 (326.7)], private health insurance [₹ 9567 (869.3)] and social insurance schemes [₹ 9,669 (636)]. It is to be noted that state sponsored schemes also comprise of patients insured through AB-PMJAY as well as state-specific schemes.

**Sources of financing out-of-pocket expenditure**

We found that nearly 16.2% patients borrowed the money from relatives/friends without interest while 4.1% borrowed with interest, and 2.2% sold their assets to meet the health expenditure for non-hospitalised treatment. For hospitalization, 12.7% patients borrowed money from relatives and friends, 3.5% borrowed with interest and 1.6% sold their assets. (Figure 1)


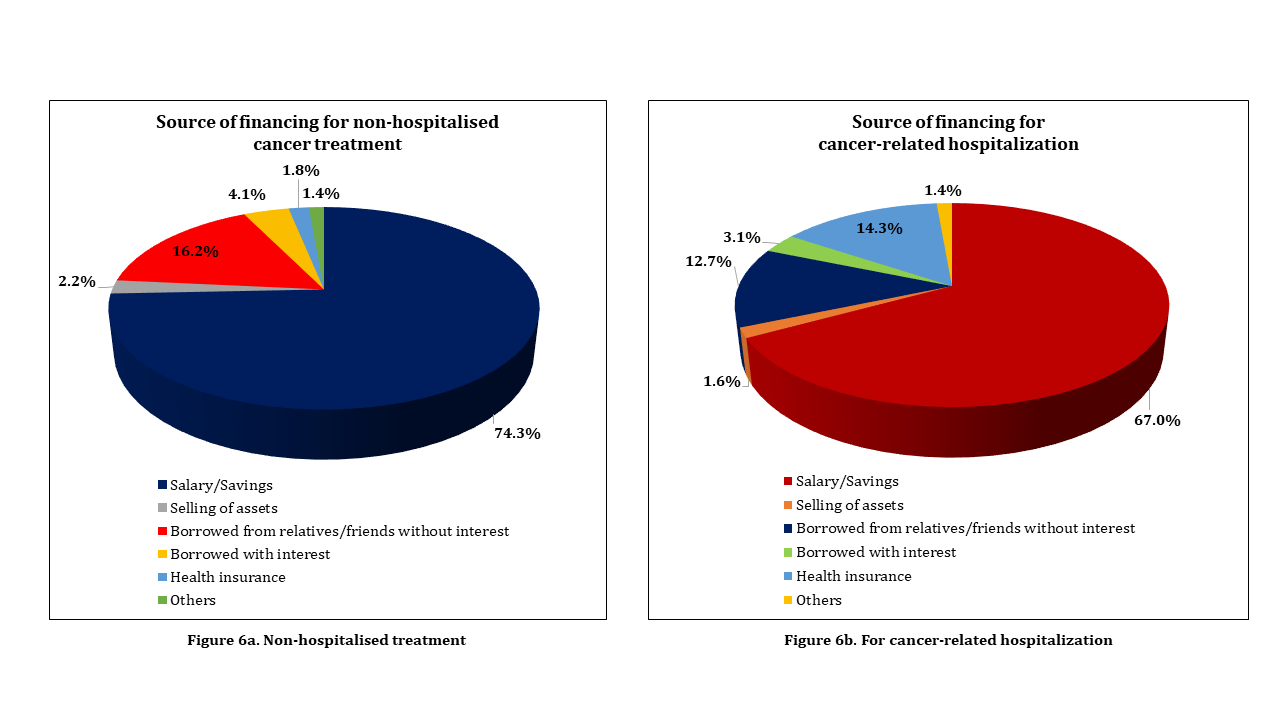


**Figure 1: Source of financing for cancer treatment**

**Table 1: Association between socio-demographic characteristics and out-of-pocket expenditure (OOPE) for outpatient (outpatient) cancer treatment**

| Sociodemographic characteristics | Number of patients, N (%) | Mean OOPE in ₹ (SE) | Median OOPE in ₹ (IQR) | p-value |
| --- | --- | --- | --- | --- |
| Age groups (in years) |  |  |  |  |
| 0-15 | 311 (3.2%) | 8334 (648.3) | 5000 (8220) | 0.018 |
| 16-30 | 778 (7.9%) | 7663 (411.3) | 4000 (7150) |  |
| 31-45 | 2559 (26.1%) | 7537 (287.6) | 3350 (6900) |  |
| 45-60 | 3965 (40.5%) | 7977 (237.0) | 3700 (7600) |  |
| Above 60 | 2174 (22.2%) | 8900 (293.6) | 4115 (8900) |  |
| Gender |  |  |  |  |
| Male | 4078 (41.7%) | 8907 (227.5) | 4400 (8890) | 0.001 |
| Female | 5709 (58.3%) | 7444 (184.7) | 3400 (6900) |  |
| Area of Residence |  |  |  |  |
| Urban | 3381 (34.5%) | 8056 (293.2) | 3100 (7500) | 0.591 |
| Rural | 6269 (64.1%) | 8079 (158.2) | 4050 (7700) |  |
| Slum | 137 (1.4%) | 6821 (776.7) | 3200 (7600) |  |
| Education |  |  |  |  |
| No education | 2124 (21.7%) | 7292 (229.5) | 3700 (6900) | <0.01 |
| Primary & Middle | 3435 (35.1%) | 7137 (205.0) | 3350 (6900) |  |
| Up to Senior Secondary | 2942 (30.1%) | 8585 (293.2) | 3969 (8410) |  |
| Graduation & above | 1286 (13.1%) | 10545 (542.8) | 4685 (10123) |  |
| Wealth Quintile |  |  |  |  |
| Poorest | 1958 (20%) | 4839 (198.2) | 2000 (4335) | <0.01 |
| Poor | 1960 (20%) | 6301 (313.1) | 3077 (5800) |  |
| Middle | 1956 (20%) | 7565 (319.3) | 4000 (7400) |  |
| Rich | 1956 (20%) | 9307 (323.4) | 4500 (8650) |  |
| Richest | 1957 (20%) | 12260 (394.5) | 6420 (12580) |  |
| Marital Status |  |  |  |  |
| Unmarried | 895 (9.1%) | 7623 (386.4) | 4000 (7160) | 0.254 |
| Married | 7823 (79.9%) | 8153 (162.9) | 3800 (7900) |  |
| Separated/Divorced | 66 (0.7%) | 5204 (831.4) | 2490 (6970) |  |
| Widow/Widower | 1003 (10.2%) | 7845 (478.4) | 3200 (6520) |  |
| Health insurance |  |  |  |  |
| AB PM-JAY* | 1009 (10.3%) | 7989 (326.7) | 4200 (7700) | <0.01 |
| State Government Sponsored^#^ | 3230 (33%) | 5840 (174.3) | 2900 (5100) |  |
| Social Insurance Scheme | 568 (5.8%) | 9669 (636.0) | 4199 (10090) |  |
| Private Health Insurance | 369 (3.8%) | 9567 (869.3) | 2941 (8611) |  |
| Philanthropist/NGO | 618 (6.3%) | 4164 (315.0) | 1500 (3375) |  |
| Not covered | 3993 (40.8%) | 10092 (278.5) | 5200 (9800) |  |
| Total | **9787** | **8053 (143.7)** | **3730 (7600)** |  |

*^*^AB PM-JAY-Ayushman Bharat Pradhan Mantri Jan Aarogya Yojana, ^#^State government sponsored category includes patients enrolled in AB-PMJAY and other state health insurance schemes*

## Mean direct OOPE on outpatient treatment and its association with clinical characteristics of cancer patients

Majority (78%) of the patients were diagnosed with solid cancers and 21.5% with haematological cancers (Table 2). For the rest of the patients (0.4%), the primary site of cancer was not known. The highest proportion (22.1%) of cancer patients were in stage III, followed by stage IV (16%), stage II (12.1%), and stage I (4.2%). A small proportion (0.04%) of cancer patients were diagnosed with carcinoma in situ. At the time of recruitment, 44% of the cancer patients had received chemotherapy since their last visit to the hospital, 3.5% had received radiotherapy, 5.3% had undergone surgery, 2.4% received hormone therapy, 2.4% were on palliative care, and 9.3% received a combination of the therapies mentioned above. The rest of the patients had visited the hospital for diagnostic purpose (1%), maintenance therapy (1.8%), and for receiving other treatments (17%; blood transfusion, follow up, under observation, supportive care, etc.).

Per-visit mean OOPE incurred on outpatient treatment was found to be ₹ 8,053 (143.7). The results of the stratified analysis showed that clinical characteristics, such as type of cancer, type of treatment, stage of cancer, response to treatment, and adverse effect of treatment were significantly associated (p<0.001) with OOPE incurred on outpatient treatment. No significant association was found between OOPE incurred and the line of treatment received (p>0.05).

Among different cancer types, OOPE was found to be the highest for haematological cancers [₹ 8,728 (282.9)] followed by solid cancer [₹ 7,882 (167)], and lowest for cancers of unknown primary site [₹ 6,385 (866.4)]. Among different types of treatments received since last visit, OOPE on diagnostics was found to be highest [₹ 14,653 (1455.8)] followed by surgery [₹ 9,420 (282.9)], maintenance therapy [₹ 9,048 (904.3)], radiotherapy [₹ 9,016 (772.1)], chemotherapy [8,491 (235.6)], palliative care [₹ 8,387 (751)], and combination therapy [₹ 6,637 (332.8)]. Among patients with progressive disease, mean OOPE was found to be higher [₹ 7,737 (521.8)] than patients in progression-free survival health state [₹ 5,731 (315.9)].

**Table 2: Association between clinical characteristics and out-of-pocket expenditure (OOPE) for outpatient cancer treatment**

| Clinical characteristics | Number of patients, N (%) | Mean OOPE in ₹ (SE) | Median OOPE in ₹ (IQR) | p-value |
| --- | --- | --- | --- | --- |
| Type of cancer |  |  |  |  |
| Solid | 7618 (78%) | 7882 (167.0) | 3500 (7500) | 0.041 |
| Haematological | 2101 (21.5%) | 8728 (282.9) | 4400 (8400) |  |
| Cancer of Unknown Primary Site | 42 (0.4%) | 6385 (866.4) | 5235 (6700) |  |
| Type of treatment |  |  |  |  |
| Chemotherapy | 4304 (44%) | 8491 (235.6) | 4200 (7521) | <0.01 |
| Radiotherapy | 347 (3.5%) | 9016 (772.1) | 4000 (8100) |  |
| Palliative care | 236 (2.4%) | 8387 (751) | 4000 (7335) |  |
| Surgery | 519 (5.3%) | 9420 (687.6) | 3850 (9080) |  |
| Combination therapy* | 913 (9.3%) | 6637 (332.8) | 3280 (6698) |  |
| Hormone Therapy | 238 (2.4%) | 4244 (851.8) | 1300 (2600) |  |
| Maintenance Therapy | 179 (1.8%) | 9048 (904.3) | 5500 (7310) |  |
| Diagnostics | 97 (1.0%) | 14653 (1455.8) | 9700 (15407) |  |
| Others | 1666 (17%) | 5412 (236.6) | 2178 (4900) |  |
| No Information | 1288 (13.2%) | 10210 (462.8) | 5220 (11305) |  |
| Cancer Stage |  |  |  |  |
| Carcinoma in Situ | 4 (0.41%) | 4896 (1849.0) | 4012 (5691) | <0.01 |
| Stage I | 413 (4.2%) | 5538 (602.2) | 2400 (5020) |  |
| Stage II | 1181 (12.1%) | 7229 (369.7) | 3350 (6080) |  |
| Stage III | 2165 (22.1%) | 7639 (375.9) | 3200 (7060) |  |
| Stage IV | 1564 (16%) | 9565 (384.1) | 5000 (9400) |  |
| No Information | 4460 (45.6%) | 8178 (186.8) | 4000 (8005) |  |
| Response to Treatment |  |  |  |  |
| Progression Free Survival | 2402 (24.5%) | 5731 (315.9) | 2197 (4600) | <0.01 |
| Progressive Diseases | 450 (4.6%) | 7737 (521.8) | 3775 (8100) |  |
| Ongoing | 5394 (55.1%) | 8514 (179.2) | 4215 (7800) |  |
| Treatment not started | 1334 (13.6%) | 11232 (459.4) | 6250 (12500) |  |
| No Information | 207 (2.1%) | 3209 (313.3) | 1762 (3685) |  |
| Line of Treatment |  |  |  |  |
| First Line | 6817 (69.7%) | 7558 (151.1) | 3500 (6950) | 0.181 |
| Second Line | 1146 (11.7%) | 8234 (595.6) | 3655 (7570) |  |
| Third Line | 163 (1.7%) | 9657 (1229.8) | 4880 (9080) |  |
| Fourth Line | 20 (0.2%) | 9145 (2909.6) | 4925 (9880) |  |
| Others* | 5 (0.1%) | 3786 (1747.3) | 2030 (1400) |  |
| Treatment not started | 1334 (13.6%) | 11232 (459.4) | 6250 (12500) |  |
| No Information | 302 (3.1%) | 3638 (339.8) | 1668 (3657) |  |
| Adverse Effects |  |  |  |  |
| Without Adverse Effects | 564 (5.8%) | 3354 (273.2) | 1300 (2348) | <0.01 |
| With Adverse Effects | 5145 (52.6%) | 8379 (184.4) | 4000 (7820) |  |
| No Information | 4078 (41.7%) | 8293 (250.1) | 3800 (8020) |  |
| Total | **9787** | **8053 (143.7)** | **3730 (7600)** |  |

**Combination therapy: Chemotherapy + Radiotherapy, Surgery + Radiotherapy, Surgery + Chemotherapy, Surgery + Chemotherapy + Radiotherapy, Others-Fifth/sixth line of treatment*

## Out-of-pocket expenditure on hospitalisation

The estimated annual mean direct OOPE on hospitalisation (all episodes in last one year) was ₹ 57,553 (2953.4). The results of the stratified analysis to determine the association between OOPE on hospitalisation and socio-demographic and clinical characteristics of cancer patients are given in Table 3. Among the hospitalised cancer patients, 55.1% were hospitalised in public and 44.9% in private hospitals. Majority (57.6%) were hospitalised for more than five days. Overall, it was found that the area of residence, level of education, wealth quintile, health insurance coverage, type of hospital, and duration of hospital stay were significantly (p<0.001) associated with OOPE incurred by patients on hospitalisation. However, the difference in the OOPE incurred was statistically insignificant (p>0.05) for variables like age, gender, and marital status.

Highest OOPE was concentrated among patients belonging to urban areas [₹ 75,034 (6406.6)], followed by rural areas [₹ 48,257 (2925.8)] and slums [₹ 36,127 (6763.0)]. OOPE showed a declining trend with decreasing level of education among cancer patients. Patients with graduation or higher level of education were found to have incurred the highest OOPE [₹ 1,11,723 (14673.5)] followed by those with up-to senior secondary education [₹ 61,058 (4266.5)], primary and middle school education [₹ 41,614 (3644.4)], and no education [₹ 42, 800 (4379.5)]. Patients belonging to the richest wealth quintile incurred the highest OOPE [₹ 84,400 (6446.8)] on hospitalisation followed by the rich [₹ 63,216 (7916.3)], middle [₹ 56,946 (8102.9)], poor [₹ 43,832 (4043.2)], and the poorest income quintiles [32.250 (3476.7)]. Health insurance coverage also showed a significant impact on OOPE wherein the patients covered under various insurance schemes like state-sponsored health insurance schemes [₹ 40,462 (3406.3)], AB-PMJAY [₹ 32,824 (3872.5)], social insurance schemes [₹ 71,258 (17246.8)] and private health insurance [₹ 96,871 (15203.3)] were found to incur lesser expenditure on cancer treatment as compared to those who were not covered [₹ 81,596 (6090.6)]. Considering the type of hospital, cancer patients incurred a significantly higher OOPE in private hospitals [₹ 79, 342 (5382.8)] than in public hospitals [₹ 39,784 (2945.4)]. It was observed that OOPE increased with increasing duration of hospital stay; the highest OOPE [₹ 76,273 (4796.3)] was incurred by patients whose duration of hospitalisation exceeded five days.

## Mean direct OOPE due to hospitalization and its association with socio-demographic and clinical characteristics of cancer patients

OOPE was also found to be highest among patients above 60 years of age [₹ 64,804 (SE-5,502)]. Male patients incurred higher OOPE [₹ 56833(3150.5)] than female patients [₹ 53612(2168.3)].

**Table-3: Association between direct out-of-pocket expenditure (OOPE) on hospitalisation and sociodemographic and clinical characteristics of patients**

| Category | Number of patients, N (%) | Mean OOPE in ₹ (SE) | Median OOPE in ₹ (IQR) | p-value |
| --- | --- | --- | --- | --- |
| Age groups (in years) |  |  |  |  |
| 0-15 | 232 (4.6%) | 32681(3266.5) | 15000(30075) | 0.007 |
| 16-30 | 500 (9.8%) | 47448(4447.7) | 15148(45034) |  |
| 31-45 | 1296 (25.4%) | 53791(3802.6) | 15000(44163) |  |
| 45-60 | 2020 (39.6%) | 55331(2548.4) | 16399(51015) |  |
| Above 60 | 1047 (20.5%) | 64804(5504.2) | 20000(54508) |  |
| Gender |  |  |  |  |
| Male | 2324 (45.6%) | 56833(3150.5) | 15561(45000) | 0.388 |
| Female | 2771 (54.4%) | 53612(2168.3) | 17000(48874) |  |
| Area of Residence |  |  |  |  |
| Urban | 1972 (38.7%) | 79534(3878.9) | 30000(74000) | <0.001 |
| Rural | 3046 (59.8%) | 39994(1772.5) | 12141(31575) |  |
| Slum | 77 (1.5%) | 25654(4652.4) | 8211(22606) |  |
| Education |  |  |  |  |
| No education | 972 (19.1%) | 34104(2811.2) | 9976(26212) | <0.001 |
| Primary & Middle | 1685 (33.1%) | 38660(2268.1) | 12600(36409) |  |
| Up to Senior Secondary | 1562 (30.7%) | 56228(2664.2) | 20130(53304) |  |
| Graduation & above | 876 (17.2%) | 107898(7830.3) | 40258(100218) |  |
| Wealth Quintile |  |  |  |  |
| Poorest | 1019 (20%) | 36074(2208.7) | 10000(40958) | <0.001 |
| Poor | 1019 (20%) | 49601(2967.3) | 17000(45725) |  |
| Middle | 1019 (20%) | 56271(5451.7) | 15000(43077) |  |
| Rich | 1020 (20%) | 52157(4312.9) | 15000(42186) |  |
| Richest | 1018 (20%) | 81332(4836.6) | 26762(70000) |  |
| Marital Status |  |  |  |  |
| Unmarried | 591 (11.6%) | 47184(3989.7) | 17000(43605) | 0.369 |
| Married | 3986 (78.2%) | 56627(2161.6) | 17000(46758) |  |
| Separated/Divorced | 37 (0.7%) | 41251(16259.6) | 6635(19836) |  |
| Widow/Widower | 481 (9.4%) | 53035(6419.3) | 15000(51281) |  |
| Health insurance |  |  |  | <0.001 |
| AB-PMJAY | 1931 (37.9%) | 75195(3787.5) | 25736(62595) |  |
| State Government Sponsored^#^ | 634 (12.4%) | 24883(2318.6) | 10000(16452) |  |
| Social Insurance Scheme | 1461 (28.7%) | 35101(2227.1) | 10000(34316) |  |
| Private Health Insurance | 421 (8.3%) | 75176(9120.3) | 22658(70334) |  |
| Philanthropist | 331 (6.5%) | 92713(6894.4) | 50291(87653) |  |
| Not covered | 317 (6.2%) | 19061(2437.5) | 3762(19289) |  |
| Type of hospital |  |  |  | <0.001 |
| Public | 2236 (43.9%) | 33294(2028.4) | 11346(25000) |  |
| Semi-Private | 1630 (32%) | 66676(2968.3) | 27885(57692) |  |
| Private | 1229 (24.1%) | 79342(5382.8) | 20000(90000) |  |
| Duration of hospitalization (days) |  |  |  |  |
| 1 | 592 (11.6%) | 50226(2920) | 31882(41451) | <0.001 |
| 2 | 477 (9.4%) | 30639(2396.6) | 8892(30695) |  |
| 3 | 566 (11.1%) | 30652(2079.4) | 10000(34238) |  |
| 4 | 584 (11.5%) | 29236(2258) | 9016(24431) |  |
| 5 | 602 (11.8%) | 39270(4017.5) | 11286(29870) |  |
| >5 | 2274 (44.6%) | 78376(3784.5) | 22679(72914) |  |
| Total | **5095** | **55081(1858.9)** | **16382(46348)** |  |

*^*^AB PM-JAY-Ayushman Bharat Pradhan Mantri Jan Aarogya Yojana, ^#^State government sponsored category includes patients enrolled in AB-PMJAY and other state-specific health insurance schemes*

## Determinants of out-of-pocket expenditure due to outpatient and hospitalized treatment

The study also determined the factors affecting OOPE due to cancer-related hospitalization (Table 4) and outpatient treatment (Table 5).

## Outpatient treatment

The results of the regression analysis showed that OOPE incurred by female patients (B= -609.49, p<0.001) was significantly lower as compared to male patients. In addition, the OOPE incurred by patients with up to senior secondary (B = 1573.3) or graduation and above (B = 1912.82) level of education were found to be significantly higher (p<0.001) as compared to those with no education. Patients from the rich and richest wealth quintiles incurred significantly higher OOPE (B = 2531.89 and B = 5297.59; p<0.001) as compared to poorest income groups. Further, patients insured under different health insurance schemes (except private health insurance) were found to have incurred lower OOPE as compared to those who were not insured (B= -2010.76 for AB-PMJAY; B = -3004.54; B = -1440.55; B = -4165.63; p<0.001). Significantly higher OOPE was incurred by patients on diagnostics [B = 5810.12, p<0.001]. [Table 4]

**Table 4: Factors affecting out-of-pocket expenditure on outpatient treatment**

| Parameter | | B | Std. Error | 95% Confidence Interval | | Sig. |
| --- | --- | --- | --- | --- | --- | --- |
|  |  |  |  | **Lower** | **Upper** |  |
| (Intercept) | | 6870.01 | 802.68 | 5296.79 | 8443.23 | 0.000 |
| Age | | 16.57 | 10.06 | -3.15 | 36.28 | 0.100 |
| Gender Ref Male | Female | -609.49 | 308.80 | -1214.74 | -4.25 | 0.048 |
| Education Ref No Education | Primary & Middle | 547.75 | 404.30 | -244.67 | 1340.17 | 0.176 |
|  | Up to Senior Secondary | 1573.31 | 420.41 | 749.32 | 2397.29 | <0.01 |
|  | Graduation & above | 1912.82 | 524.81 | 884.21 | 2941.43 | <0.01 |
| Wealth Quintile (Reference- Poorest) | Poor | 556.71 | 459.08 | -343.06 | 1456.49 | 0.226 |
|  | Middle | 1088.85 | 480.42 | 147.25 | 2030.45 | 0.024 |
|  | Rich | 2531.89 | 488.94 | 1573.60 | 3490.19 | <0.01 |
|  | Richest | 5297.59 | 487.55 | 4342.00 | 6253.17 | <0.01 |
| Health Insurance (Reference -Not Covered) | AB PM-JAY | -2010.76 | 547.03 | -3082.91 | -938.60 | <0.01 |
|  | State Sponsored | -3004.54 | 355.34 | -3701.00 | -2308.08 | <0.01 |
|  | Social Insurance Scheme | -1440.55 | 667.73 | -2749.28 | -131.83 | 0.031 |
|  | Private Health Insurance | 110.61 | 799.79 | -1456.95 | 1678.16s | 0.890 |
|  | Philanthropist | -4165.63 | 616.50 | -5373.94 | -2957.31 | <0.01 |
| Type of Cancer (Reference-Solid) | Haematological | 91.79 | 392.22 | -676.94 | 860.53 | 0.815 |
|  | CUPS | -1483.27 | 2336.62 | -6062.96 | 3096.42 | 0.526 |
| Type of Treatment (Reference- Chemotherapy) | Radiotherapy | 467.38 | 756.20 | -1014.74 | 1949.51 | 0.537 |
|  | Palliative care | -58.63 | 898.86 | -1820.37 | 1703.10 | 0.948 |
|  | Surgery | 860.37 | 630.40 | -375.18 | 2095.92 | 0.172 |
|  | Combination Therapy | -1941.23 | 497.05 | -2915.44 | -967.02 | <0.01 |
|  | Maintenance Therapy | -1475.90 | 1052.71 | -3539.18 | 587.38 | 0.162 |
|  | Diagnostic | 5810.12 | 1377.12 | 3111.01 | 8509.22 | <0.01 |
|  | Hormone Therapy | -3134.94 | 902.25 | -4903.31 | -1366.56 | 0.001 |
|  | Others | -2993.18 | 393.06 | -3763.56 | -2222.80 | <0.01 |

*^*^AB PM-JAY-Ayushman Bharat Pradhan Mantri Jan Aarogya Yojana*

## Hospitalisation

Mean OOPE incurred by patients having an education of graduation and above was found to be significantly higher (B = 46638.6) as compared to those with no education. Further, OOPE incurred by patients belonging to middle (B = 28235.5), rich (B = 20374.6), and richest (B = 32130.2) wealth quintiles were significantly higher (p<0.001) as compared to those from the poorest income groups. In addition, patients insured under different health insurance schemes (except private health insurance) were found to have incurred lower OOPE as compared to those who were not insured (B= -28797.2 for AB-PMJAY; B = -48108.3 for state government sponsored schemes; B = -13504.5 for social insurance schemes; B = -68930.8 for philanthropists/NGOs; p<0.001). For patients covered under private health insurance schemes, the OOPE incurred was higher (B =410.7) than those who are not covered. However, the results were not statistically significant (p=0.957). Significantly higher OOPE was incurred by patients who sought hospitalization in private hospitals [B = 50613.9, p<0.001] as compared to public hospitals. Significant increase in OOPE was found with increase in duration of hospitalisation (B = 2924.9). [Table 5]

**Table 5: Factors affecting out-of-pocket expenditure due to cancer-related hospitalization**

| Parameter | | B | Std. Error | 95% Confidence Interval | | Sig. |
| --- | --- | --- | --- | --- | --- | --- |
|  |  |  |  | **Lower** | **Upper** |  |
| (Intercept) | | -9817.2 | 9812.4 | -29049.2 | 9414.7 | 0.317 |
| Age | | 280.5 | 113.9 | 57.2 | 503.8 | 0.014 |
| Area of Residence (Ref- Urban) | Rural | -13190.6 | 3982.9 | -20997.0 | -5384.1 | 0.001 |
|  | Slum | -26987.0 | 14524.0 | -55453.6 | 1479.6 | 0.063 |
| Education (Ref- No Education) | Primary & Middle | -231.5 | 5016.7 | -10064.0 | 9601.1 | 0.963 |
|  | Up to Senior Secondary | 11422.7 | 5194.8 | 1241.1 | 21604.3 | 0.028 |
|  | Graduation & above | 46638.6 | 6214.0 | 34459.2 | 58817.9 | 0.000 |
| Wealth Quintile (Ref- Poorest) | Poor | 16264.2 | 5559.9 | 5367.1 | 27161.4 | 0.003 |
|  | Middle | 28235.5 | 5753.9 | 16958.2 | 39512.8 | 0.000 |
|  | Rich | 20374.6 | 5754.0 | 9097.0 | 31652.1 | 0.000 |
|  | Richest | 32130.2 | 5850.4 | 20663.7 | 43596.7 | 0.000 |
| Health Insurance (Ref-Not Covered ) | ABPMJAY | -28797.2 | 5790.3 | -40145.9 | -17448.5 | 0.000 |
|  | State Sponsored | -48108.3 | 4558.8 | -57043.3 | -39173.2 | 0.000 |
|  | Social Insurance Scheme | -13504.5 | 6658.7 | -26555.2 | -453.8 | 0.043 |
|  | Private Health Insurance | 410.7 | 7632.8 | -14549.4 | 15370.7 | 0.957 |
|  | Philanthropist | -68930.8 | 7818.6 | -84255.0 | -53606.6 | 0.000 |
| Type of Hospital (Ref- Public) | Semi-Private | 46472.3 | 4443.4 | 37763.5 | 55181.2 | 0.000 |
|  | Private | 50613.9 | 4806.3 | 41193.8 | 60034.0 | 0.000 |
| Duration of stay during hospitalization | | 2924.9 | 158.1 | 2615.0 | 3234.8 | 0.000 |

*^*^AB PM-JAY-Ayushman Bharat Pradhan Mantri Jan Aarogya Yojana*

## Health care burden due to cancer, stratified according to primary cancer site

A stratified analysis was also done to ascertain the OOPE incurred based on the primary site of cancer for both hospitalisation and outpatient treatment. In addition, the health care burden (defined as OOPE as a proportion of consumption expenditure) was computed for different categories of cancers based on primary site as shown in Table 6.

**Table 6: Average OOPE on cancer treatment and health care burden by cancer type**

| **Category of Cancer** | **Hospitalisation** | | | **Non-hospitalised treatment** | | |
| --- | --- | --- | --- | --- | --- | --- |
|  | **OOPE (INR)** | **Annual Consumption Expenditure (INR)** | **Health care burden* (%)** | **OOPE (INR)** | **Annual Consumption Expenditure (INR)** | **Health care burden*(%)** |
| **Bladder cancer** | 43594 | 251059 | 17.4% | 260961 | 211755 | 123% |
| **Bone cancer** | 35662 | 226703 | 15.7% | 314139 | 209291 | 150% |
| **Brain and other nervous system cancer** | 61877 | 236062 | 26.2% | 234076 | 225272 | 104% |
| **Breast cancer** | 49633 | 231928 | 21.4% | 231857 | 204626 | 113% |
| **Cancer of unknown primary site (CUPS)** | 16743 | 244304 | 6.9% | 227591 | 207876 | 109% |
| **Cervical and Uterine cancer** | 32174 | 238802 | 13.5% | 210926 | 216311 | 98% |
| **Colorectal cancer** | 83942 | 252305 | 33.3% | 297941 | 211213 | 141% |
| **Head and Neck cancer** | 36394 | 236256 | 15.4% | 240891 | 214235 | 112% |
| **Oral cancer** | 45657 | 229146 | 19.9% | 257335 | 223774 | 115% |
| **Kidney and Ureter Cancer** | 96195 | 275916 | 34.9% | 363673 | 277147 | 131% |
| **Leukaemia** | 95877 | 245156 | 39.1% | 227919 | 225165 | 101% |
| **Lung cancer** | 55178 | 254094 | 21.7% | 389255 | 246733 | 158% |
| **Lymphoma** | 55792 | 257139 | 21.7% | 348024 | 252856 | 138% |
| **Multiple Myeloma** | 62455 | 261297 | 23.9% | 373297 | 278326 | 134% |
| **Ovarian cancer** | 56593 | 232221 | 24.4% | 211315 | 220665 | 96% |
| **Pancreatic and Biliary cancer** | 65954 | 230107 | 28.7% | 294059 | 222886 | 132% |
| **Prostate cancer** | 49770 | 238359 | 20.9% | 399676 | 211910 | 189% |
| **Penile cancer** | 17210 | 203048 | 8.5% | 254902 | 209514 | 122% |
| **Skin cancer** | 22535 | 211564 | 10.7% | 280319 | 204716 | 137% |
| **Soft tissue tumours** | 38024 | 185530 | 20.5% | 202093 | 204516 | 99% |
| **Testicular cancer** | 20156 | 239612 | 8.4% | 181725 | 223648 | 81% |
| **Upper GI tract cancer** | 41184 | 214622 | 19.2% | 278137 | 204764 | 136% |
| **Other haematological cancers** | 98499 | 271978 | 36.2% | 372048 | 263982 | 141% |
| **Other cancers*** | 68090 | 224006 | 30.4% | 249651 | 210082 | 119% |
| **Total** | 55081 | 238134 | 23.1% | 266726 | 221984 | 120% |
